# Probabilistic Approach for Assessing the Occupational Risk of Olfactometric Examiners: Methodology Description and Application to Real Exposure Scenario

**DOI:** 10.3390/toxics12110784

**Published:** 2024-10-29

**Authors:** Elisa Polvara, Andrea Spinazzè, Marzio Invernizzi, Andrea Cattaneo, Domenico Maria Cavallo, Selena Sironi

**Affiliations:** 1 Politecnico di Milano, Department of Chemistry, Materials and Chemical Engineering “Giulio Natta”, Piazza Leonardo da Vinci 32, 20133 Milano, Italy; elisa.polvara@polimi.it (E.P.); selena.sironi@polimi.it (S.S.); 2 Department of Science and High Technology DiSAT, Università degli Studi dell’Insubria, Via Valleggio 11, 22100 Como, Italy; andrea.spinazze@uninsubria.it (A.S.); andrea.cattaneo@uninsubria.it (A.C.); domenico.cavallo@uninsubria.it (D.M.C.)

**Keywords:** occupational exposure, Monte Carlo analysis, olfactometric panelists, dynamic olfactometry, odorous emissions

## Abstract

Human examiners, known as panelists, are exposed to an unknown occupational exposure risk while determining odor concentration (C_od_) using dynamic olfactometry. In the literature, a few papers, based on a deterministic approach, have been proposed to establish this occupational risk. As a result, the purpose of this study is to develop and apply a probabilistic approach, based on the randomization of exposure parameters, for assessing and evaluating the occupational exposure risk among olfactometric examiners. In this methodology, the risk is assessed by computing the hazard index (HI) and inhalation risk (IR) to determine the non-carcinogenic and carcinogenic risks. To randomize the exposure parameters, a Monte Carlo simulation was described and then applied in real exposure scenario to establish the exposure risk in terms of probability. Therefore, a one-year survey of the working activity of olfactometric examiners of *Laboratorio Olfattometrico* of Politecnico di Milano university was conducted. Based on this data collection (exposure parameters and chemical data, divided according to sample categories), a randomized exposure scenario was constructed to estimate the probability and cumulative distribution function of risk parameters. Different distributions were obtained for different industrial samples categories and were compared with respect to acceptability criteria (the value of HI and IR at 95th percentile of distribution). The elaboration provided evidence that negligible non-carcinogenic and carcinogenic risks are associated with the panelists’ activity, according to an entire annual dataset. The application of probabilistic risk assessment provides a more comprehensive and effective characterization of the general exposure scenario for olfactometric examiners, surpassing the limitations of a deterministic approach. This method can be extended to future exposure scenarios and enables the selection of the most effective risk management strategies to protect the health of olfactometric examiners.

## 1. Introduction

The odor potential of atmospheric emissions from industrial plants is commonly measured by dynamic olfactometry [1,2,3,4]. This technique allows the quantification of odor concentration (in terms of European odor units per cubic meter, ou_E_/m^3^) of gaseous emissions collected in sampling bags. Dynamic olfactometry, standardized by EN 13725 [5], is a sensorial technique, involving human examiners, also called panelists. To quantify odor concentration, the gaseous samples are diluted with neutral air and presented to panelists in rising concentrations. Thus, during the analysis, even at highly diluted concentrations, panelists sniff the compounds present in emission samples and any potentially hazardous chemicals that may be present in the samples being analyzed [6,7].

Despite the relevance of this issue and the high number of olfactometric laboratories in the world, this topic still appears to be underdeveloped both at the regulatory level and within the literature.

The few scientific studies about this topic are based on a deterministic risk assessment approach which presents a degree of uncertainty due to the high variability associated with the activity performed by olfactometric workers and laboratories. The type and the numerosity of analyzed samples and the panelists’ exposure modes (i.e., frequency, intensity, duration of the exposure to hazardous chemicals in samples) are strictly correlated with the working activity of every single panelist and of every single olfactometric laboratory. In addition, odorous samples are typically characterized by great variability, in terms of the concentration and nature of compounds: the application of a deterministic approach for risk assessment would not allow to consider these variabilities, therefore making it difficult to extend the evaluation (with a prospective approach) to a wider range of cases when assessing occupational risk for this category of workers.

Furthermore, the activity of the panelists does not take the form of a full-time activity. To prevent nose fatigue, olfactometric panelists often work in daily periods of 1 or 2 h, and the examiner is only called for a maximum of one analysis session per day. Additionally, each sample’s single presentation lasts a few seconds. Therefore, exposure to hazardous chemicals during dynamic olfactometry typically results in short-term, intermittent exposure to a mixture of chemicals with widely varying exposure intensities.

This exposure scenario is not comparable with the typical paradigm of occupational exposure and the classical risk management hierarchy of controls (i.e., substitution in the production of chemicals by non-toxic alternatives, engineering controls, isolating people from the hazard, and the use of personal protective equipment) [8] cannot be adopted in this exposure scenario. In addition, the classical technologies adopted in the monitoring of air pollutants in the occupational setting are not applicable to the risk assessment of olfactometric assessors [9,10]. All these issues clearly suggest that it could be difficult to verify compliance with occupational exposure limits defined for occupational chemical risk assessment within the framework of occupational health and safety (OHS) policies and practices.

The implementation and application of a probabilistic approach for dynamic olfactometry panelists’ risk assessment, focusing on their real-scale occupational exposure scenarios, appears a promising solution to overcome the abovementioned criticalities [11,12], since the stochastic approach allows the introduction of randomness derived from the variability in the source data into the exposure assessment and risk characterization phase of the risk assessment process.

Thus, this paper aims to propose a new probabilistic risk assessment methodology, and to apply it to a real exposure scenario, to estimate the occupational risk for olfactometric examiners.

To reach this goal, firstly, this study will construct and present the probabilistic approach to assess occupational risk for olfactometric examiners. After that, the described methodology will be applied to investigate the occupational exposure risk for panelists working at *Laboratorio Olfattometrico* of Politecnico di Milano (PoliMI), Milan, Italy. From this data collection, a randomization of exposure parameters was conducted to determine the overall exposure scenario, to consider the total amount of analyzed olfactometric samples, even though only a consistent portion of the olfactometric samples have been chemically characterized, and to describe a general exposure scenario for olfactometric examiners.

The probabilistic method aims to provide a robust evaluation of occupation risk associated with olfactometric analyses, compared to a deterministic methodology.

## 2. State of the Art

### 2.1. Olfactometric Analysis

Dynamic olfactometry is a sensorial technique that uses dilution equipment (an olfactometer) to present odorous samples to a panel of olfactometric examiners. The odor concentration, expressed in European odor units per cubic meter (ou_E_/m^3^), is the number of dilutions with neutral air necessary to raise the sample concentration to the concentration needed to detect odors [13]. The selection procedure for examiners is described in detail in the standard. However, it is emphasized that these examiners are selected based on their response to a standard compound (*n*-Butanol).

The analysis is conducted by presenting the odorous sample to the panel at increasing concentrations using the olfactometer until the panel members are able to perceive a difference from the neutral reference air.

To determine the odor concentration, the odorous air flows through predilution units and is mixed with neutral air (with an exponential trend ratio of 1:2^n^). The initial dilution (i.e., the first presentation of the sample to the examiners) must be very high, so that the human nose cannot perceive the sample: the initial non-detectability of the sample must be ensured to be able to register the “step-response” of the perception. In the next dilution steps, the sample dilution decreases (i.e., the concentration of the presented odorous sample increases). When the examiners perceive an odor different from neutral air, they press the button to register the change. Every examiner needs to respond twice for the two consecutive presentations, to confirm his/her evaluation: this means that, in the confirmation step, the presentation concentration is twice the perception one. The single round finishes when all the assessors respond two times to the odor presentation. To improve the precision of the measurement, multiple presentations of dilution series (also defined as a *round*) of the single sample must be conducted. The minimum number of single odor threshold values, *Z_ITE_*, necessary to obtain the odor concentration, is 12. Therefore, the number of rounds (N_R_) depends on the number of involved examiners: generally, at least 4 examiners are involved in the analyses, and at least 3 measurement rounds are carried out to reach the 12 (3 values of odor threshold x 4 assessors) Z_ITE_ necessary for EN 13725 analysis.

At the end of the rounds, it is possible to obtain the odor concentration (C_od_, expressed in ou_E_/m^3^): it is calculated as the geometric mean of at least 12 odor detection threshold values multiplied by a factor depending on the olfactometer dilution factor (the square root of exponential dilution step, i.e., $\sqrt{2}$).

The standard EN 13725, in Section 9.6.1.3—*Retrospective screening of panel members after each measurement* [5], allows a specific variability among the examiners’ response (single Z_ITE_): this difference is schematized in the standard as ΔZ and is defined as the ratio between the individual threshold estimates Z_ITE_ and the geometric mean ${\bar{Z}}_{\mathrm{ITE}}$ of all individual threshold. The technical standard mandates that this difference must be as follows:

(1) −5 ≤∆Z≤+5

**Figure 1:**
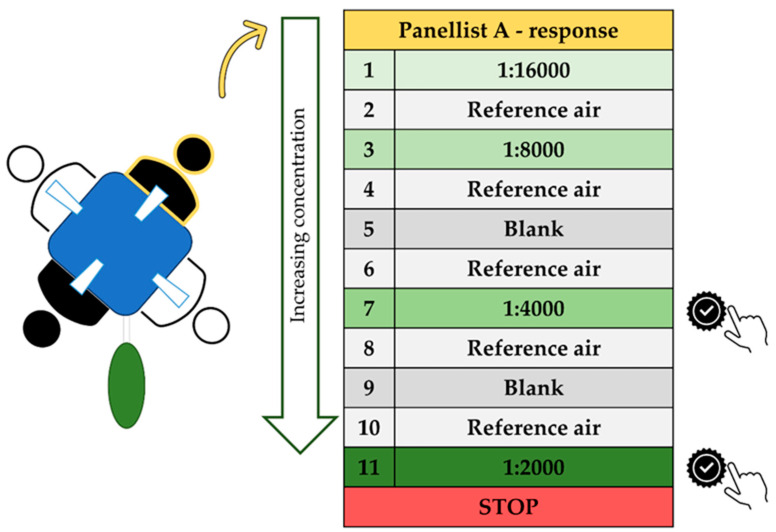
Schematization of determination of odor threshold value—single round; single examiner.

A schematization of the process of olfactometric analysis is summarized in Figure 1.

As reported in Figure 1, the sample is always alternating with reference air (e.g., only neutral air) to clean and provide a neutral air reference to the nose. In addition, the odor sample is alternated with blanks (neutral air replacing sample presentation to examiners) to check the responsiveness and reliability of examiners and verify their attention.

**Figure 2:**
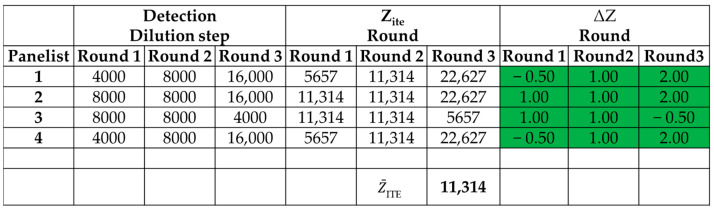
Example of response matrix. The green color shows that the obtained ΔZ values comply with the criteria prescribed by standard EN 13725.

An example of the response of four panelists (i.e., matrix of responses) for a real dynamic olfactometry analysis, with three presentation rounds, is reported in Figure 2.

During the test, the sample is presented for a few seconds. To avoid nose fatigue, the session of analysis should last 2 h at most in a single session, no limits regarding the number of samples to be analyzed are specified in the standard.

### 2.2. Risk Assessment for Olfactometric Examiners: Available Approaches

As introduced previously, the chemical risk assessment for olfactometric examiners still appears to be an insufficiently explored topic, both at the regulatory level and within the scientific and academic literature.

Focusing on the regulatory level, the European technical standard, EN 13725 [5], in its recent revision (published in February 2022), emphasizes, in Section 9.5—*Occupational safety for sampling personnel, assessors and olfactometry operators*, the potential presence of toxic compounds in odor samples [14]. According to the standard, the examined samples cannot be pretreated or filtered in any manner, to avoid sample manipulation. Due to these technical requirements, examiners are exposed to odorous compounds present directly in the source of the odor, potentially exposing them to hazardous chemicals at non-negligible concentrations. Although a paragraph in Chapter 9.5 of the standard EN 13725 (Section 9.5.3—*The panel members*) is specifically intended to deepen this issue, only general indications are provided: the standard prescribes that “there shall be no unacceptable health risk to examiners by inhaling the (diluted) odorous gas sample during the measurement process” [5]. However, as already mentioned, the standard prescribes that no manipulation of the tested gas or use of protective masks is allowed, in order not to alter the perceptibility of the odor. The only effective protective strategy recommended is the definition of a minimum dilution value (MDV), not to be exceeded during the analysis, as the panelists are exposed to increasing levels of odorous compounds during the olfactometry analysis. EN 13725 [5] underlines that a “risk assessment focused on toxicity shall be carried out by the person responsible for the health and safety” but the standard does not indicate, in its most recent revision, a harmonized and well-defined approach to assess the toxicological risk of panelists engaged in olfactometric analyses [6].

In general, a standardized method for estimating olfactometric assessors’ risk has not been defined yet, and this might cause several issues and limitations with the toxicological evaluation. Firstly, different limit values for occupational inhalation exposure are often available for the same chemical, depending on the exposure period, the exposure route, and the method by which the value was obtained from different proposing entities (this issue was also specified within the EN 13725 standard concerning hydrogen sulfide, H_2_S) [15,16,17]. For the already mentioned reasons, different authorities can propose different exposure values as a benchmark: for instance, for toluene, the occupational limit value for long-term exposure (8 h time-weighted average value) established by the European Commissions is equal to 192 mg/m^3^, while the one recommended by NIOSH 375 mg/m^3^, and the one published by ACGIH is 75 mg/m^3^ [18,19,20,21].

The EN 13725 standard does not provide any additional information about the choice of the proper occupational exposure limit values to be considered for the risk assessment of olfactometric examiners. It is worth underlining that EN 13725 defines panelists as workers, even if, as mentioned in the previous paragraph, the exposure of panelists is rather unusual when compared to other workers exposed to chemical agents. However, EN 13725 does not suggest how to consider the exposure of these workers. This deficiency strongly contributes to arbitrariness in exposure assessment and risk characterization, which may result in inconsistent risk evaluation.

The methodology for determining occupational risk for panelists was described in a limited number of scientific studies. These methods are based on the evaluation of non-carcinogenic and carcinogenic risk, by calculating common risk parameters used at an international level, namely the hazard index (HI) for non-carcinogenic risk and the inhalation risk (IR) for carcinogenic risk. These parameters were used to define the MDV not to be overcome during analyses. According to the results of the risk assessment, the definition of MDV is necessary for protecting panelists. Therefore, all the papers, discussed in detail hereafter, calculate the MDV to be applied as the only practicable risk management measure, by estimating HI and IR for panelists’ activity and by comparing the toxicological results with the olfactometer dilution step.

Discussing the different approaches available in the literature, we can divide and summarize the scientific development in three different schemes, according to the source of chemical data:

(1) The first approach can be described as *literature-based*: chemical emission data, in qualitative and quantitative terms, are collected from the literature for specific plants/sources. Through this approach [22,23,24], the MDV to be applied was determined using the highest concentration of contaminants reported in the literature. The evaluation of risk exposure, based on the literature data, allows, at the first evaluation step, a determination of the MDVs a priori, based on already available data and not having to conduct complex and time-consuming chemical characterization of real odorous samples. This is particularly important considering the limitations imposed by the standard: EN 13725 prescribes that odor samples must be analyzed by dynamic olfactometry as soon as possible after collection and, in any event, no later than 30 h after sampling. Because of this, obtaining a direct and complete chemical characterization before conducting the olfactometric analysis for each single sample and interpreting the results of these analyses for risk assessment and risk management is time-consuming and requires a considerable technical capacity, in terms of the number of instruments capable of detecting different compounds even at low concentrations, and highly qualified personnel [23,24]. Therefore, using the literature data makes it possible to quickly obtain an MDV not to be exceeded. However, an important disadvantage of using the literature data is the congruence and representativeness of the available data with the sampling activity preparatory to olfactometric analysis. The literature data available could not be comparable with the concentration potentially present in an odorous sample collected directly at the emission source: several literature studies are focused on pollutant concentration in ambient air near the sources, at the plant fence line or near sensitive receptors located in an industrial context (e.g., school, hospital, nursing home) [25,26,27,28,29]. Consequently, these concentration values are not directly correlated with the concentration observable at the emission source, where the olfactometric sample must be collected, following the standard. In addition, the use of different sampling techniques (ambient air or different capped hoods), as well as different operating conditions (e.g., sweeping air flow rate, surface temperature), may strongly influence emissions concentration [30]. The depth of these studies’ research is another critical factor: the analyses on industrial emissions focus on a few properties and/or regulated hazardous chemicals/process tracers to evaluate the risk assessment for workers or citizens. However, a comprehensive risk assessment in olfactometric activities requires a wide range of analyses, focused on the complete chemical characterization of the entire mixture. The studies available in the literature are, instead, often focused on specific target analysis. However, specific analysis may overlook the toxicological contribution of other compounds not specifically searched for.
(2) A second strategy, *permit-based,* is based on the utilization of maximum authorized concentrations for each specific emission source [31]. This strategy again proves to be very useful for obtaining immediate data, without performing detailed chemical analyses for each single compound subject to dynamic olfactometry. Even in this case, therefore, it is possible, by applying this strategy, to obtain a bias of the risk. Moreover, not all odorous sources have permitted pollutant concentration values: in fact, limits are generally imposed on stacks or channeled emissions into the atmosphere, but the odorous sources are often diffuse (e.g., heaps of stored material, liquid area, tanks) [4,32,33,34]. For these sources, there are no specific emission limits for pollutants. Therefore, a toxicological assessment for these sources cannot be conducted by this approach, and an underestimation of the global risk can occur. In addition, these values are generally averaged on an hourly basis. The olfactometric sampling is in general shorter, in the order of minutes. Considering a short sampling period, usually adopted in olfactometric sampling, we may obtain peak phenomena, not averaged over the hour. Therefore, we could sample a higher concentration of potentially hazardous pollutants and, by adopting in the risk assessment an averaged value from environmental permission, we could obtain an underestimation of the risk for some specific samples.
(3) The last approach, *analysis-based*, relies on the chemical characterization of analyzed samples [35]. By applying chemical analysis, in particular gas chromatography coupled with mass spectrometry (GC-MS), it is possible to identify and quantify the species, in general, and the volatile organic compounds (VOCs) present in the real odorous sample. In this way, it is possible to define the exposure risk associated with a single specific sample and, thus, to define its MDV not to be exceeded to ensure the safety of examiners. In addition, this analysis allows the complete and concrete characterization of VOCs present in the mixture: in this way, it is possible to obtain the complete characterization of the risk. The greatest disadvantage of this approach is the necessity to conduct this deep analysis within the 30 h between sampling and olfactometric analysis, as required by the standard. As a matter of fact, this approach, despite its robustness, is extremely time-consuming and incompatible with performing a thorough chemical speciation and toxicity investigation on every detected compound.

In addition to the variability among the sources of the chemical data, in the absence of a standardized method defined at the regulatory level, these studies employ a variety of approaches, with the possibility of obtaining different results [6].

Currently, all models reported in the literature are based only on a *deterministic* risk approach. However, the risk assessment conducted from a particular dataset of chemical data (based on the analysis of real samples or literature research) is obviously correlated with the single sample/category of samples, and this evaluation cannot be applied for all the scenarios without a specific evaluation of exposure parameters of the single examination panel [35]. This represents a huge limitation of the currently available approaches, especially related to the prediction of risk. This is particularly helpful in any occupational context, but it is especially crucial in the case of occupational exposure during olfactometric analysis. Given the wide range of concentrations (both in terms of the industrial sources to be analyzed and due to the inherent variability of an industrial process), the wide range of chemicals potentially present in the samples (different industrial process generates different types of emitted compounds) and the criticalities connected with the conduction of a deep chemical characterization in the time frame foreseen by EN 13725, it is impossible to guarantee that an olfactometric sample can be representative, given its composition. To protect the health of examiners and to adhere to the standard’s requirements, which states that samples must be analyzed within 30 h of sampling [5], it is crucial to obtain information on occupational risk that can be applied in general terms.

For this reason, overcoming the critical aspects of the deterministic approach by using a probabilistic approach, based on the randomization of real data, appears to be the best solution to evaluate the occupational exposure risk for olfactometric examiners.

### 2.3. Collection of Exposure Data: Parameters and Their Criticalities

To reach the goal of this study, the first step is the collection of exposure data, in terms of panelists working activity and chemical composition of analyzed samples. Only after this collection is it possible to apply a probabilistic approach and obtain a statistical distribution of the real exposure risk scenario.

**Figure 3:**
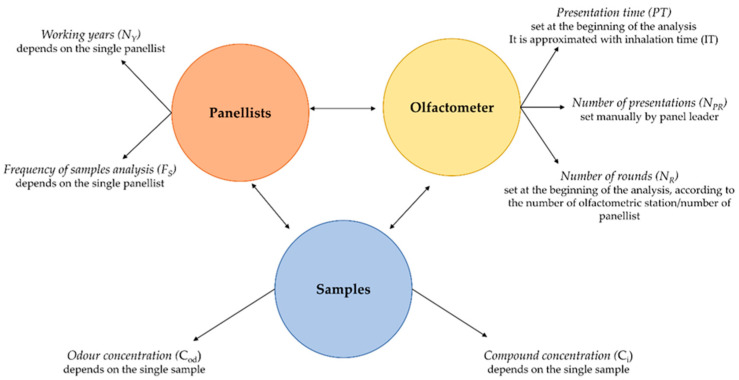
Relationship between different experimental factors and exposure parameters.

Focusing on the olfactometric examiners working activity, their exposure is strictly correlated with three different variability sources, as follows (Figure 3):

- Parameters correlated with the *olfactometer* settings;
- The chemical/odorous nature of the *samples*;
- The specific working activity of each panelist.

The parameters connected with the olfactometer set-up are as follows: (i) Presentation time (PT), equal to the seconds during which the sample is presented through the appropriate door to the examiner; (ii) number of rounds (N_R_), defined in EN 13725 as “presentation of one dilution series to all assessors”. These two parameters are defined at the beginning of the analysis because they are strictly correlated with the operative performance of the test and instrumental parameters. Indeed, the PT is generally between 2 and 3 s and it is settable at the beginning of the test on the olfactometer set-up. The N_R_, as previously discussed, relates to the number of panelists involved in the test. As a result, while these parameters may differ slightly amongst olfactometric laboratories, they always remain the same within a single laboratory. Concerning the presentation time, this approach conservatively approximates the presentation time to the inhalation time (IT), where the IT value is necessary for the calculation of the exposure dose. The last olfactometer parameter is the number of presentations (N_PR_): this parameter, as opposed to the previous one, can vary due to different factors. The N_PR_ is strictly correlated with the starting dilution value set by the panel leader (the operator conducting odor testing) and according to the sensibility of examiners to odor, which can vary within a specific ΔZ range, as described in Section 2.1.

On the contrary, the parameters connected with the panelists’ activity and the samples can vary significantly among different examiners and olfactometric laboratories.

Regarding samples, industrial odorous emissions are highly variable in terms of composition [36,37,38]. Therefore, the variability associated with the concentration of substances and the odor potential of the sample can greatly influence the exposure risk associated with this activity.

To obtain information about the nature of the analyzed samples, an exhaustive chemical characterization is necessary to determine the substances contained in the bag samples and evaluate the associated risk. Therefore, a chemical investigation must be conducted before carrying out an exposure risk assessment: in particular, the most common technique proposed to investigate the composition of odorous samples is gas chromatography coupled with mass spectrometry (GC-MS) [13,39].

To investigate not only the number of samples analyzed by dynamic olfactometry by panelists, but also the subdivision of samples in macro-categories, it could be useful to classify analyzed samples into industrial categories according to the type of production cycle. As a result of this, it is possible to quantify the samples and classify them in accordance with the activities of the single olfactometric laboratory whose activities are being monitored.

Similar diversity may be observed in the working activity of panelists, particularly by looking at the years of activity (working years, N_Y_) and the number of samples examined by each examiner during the period of survey (frequency of sample analysis, F_S_).

Therefore, for parameters connected with sample and panelist activity, using an arbitrary value (average or maximum among the observed values) can lead to a considerable error in risk estimation. Therefore, randomizing these parameters and describing the risk in terms of probability appears to be the optimal solution for estimating the risk associated with this activity.

## 3. Probabilistic Risk Assessment: Method Development

### 3.1. Exposure Concentration

During the olfactometric analyses, the examiners are exposed to odorous samples at increasing concentrations until they reach the detection threshold, defined as “for an odorant gas sample, the dilution factor at which the odorant gas has a probability of 0.5 of being detected under the conditions of the test” [5]. Therefore, the concentration of inhalation can be seen as equal to the concentration of chemicals present in the sample divided by the odor concentration (C_od_) evaluated by dynamic olfactometry. In this way, it is possible to estimate the maximum inhaled concentration by the panelists during the analysis.

However, due to the analysis requirement, the C_od_ is calculated as the geometric mean of the individual threshold estimates for the panelists: therefore, a panelist can be exposed to a higher concentration (i.e., a lower dilution step) compared with the other examiners. According to the standard, as described in Section 2.1, the differences between the panelists’ responses (ΔZ) could be equal to ±5 (Equation (1)). Considering this factor, it is possible to affirm that, potentially, a panelist can be exposed to a concentration five times higher than the odor concentration threshold, obtained as an average among all examiners involved in the analysis.

In addition, during the analysis, as described in Section 2.1, to confirm the odor perception and avoid random responds by panelists, a confirmation step is required. Therefore, the panelists are always exposed, during the validation phase, to twice the concentration of perception.

From these observations, it can be deduced that a conservative safety multiplication factor should be introduced to calculate the maximum inhalable concentration during odor concentration analysis: in particular, from the discussed observation, we defined a safety multiplication factor (k) equal to 10, according to the Equation (2).

(2) $$k=10={∆Z}_{MAX}\cdot 2$$

Therefore, the inhalation concentration (i.e., the exposure concentration, C_in,i_) to be used in toxicological assessment could be estimated as follows:

(3) $$C_{in,i}=\frac{C_{i}}{C_{od}}\cdot k$$

where C_i_ is the concentration of molecule measured in the sample by chemical analysis, expressed in mg/m^3^; C_od_ is the measured odor concentration of sample; k is the safety multiplication factor.

Contrary to approaches previously presented in the scientific literature, which used the substance concentration present in the gaseous sample and then compared it to the olfactometer’s dilution steps to obtain a MDV [7,22,31,35], Equation (3) makes it possible to estimate the risk associated with the actual inhaled concentration. It is important to emphasize that, by using these assumptions, it is possible to argue that, if compared to previously proposed approaches, a very conservative assessment is being made.

### 3.2. Risk Characterization

To determine the risk associated with the exposure to hazardous chemicals of panelists, the non-carcinogenic and carcinogenic risks were calculated according to previous studies [7,35]. Focusing on non-carcinogenic risk, the HI, which is equal to the sum of each chemical component’s hazard quotient (HQ), was used to assess the possible non-carcinogenic chronic risk, via Equation (4) [40]. In this exposure scenario, according to their activity, the occupational exposure limit (OEL) for a short exposure (15 min) can be adopted [7,35].

Usually, the non-carcinogenic risk was considered unacceptable for HI values higher than 1. Section 3.3.3 should be referred to for a more detailed discussion of the acceptability criteria considered by applying the probabilistic approach.

(4) $$HI=\sum_{i}^{N}{HQ}_{i}=\frac{C_{i}}{{OEL}_{i}}$$

In light of Equation (3), the evaluation of HI panelists’ exposure is as follows:

(5) $$HI=\sum_{i}^{N}{HQ}_{i}=\frac{C_{in,i}}{{OEL}_{i}}$$

The carcinogenic risk, generally speaking it can be calculated via the estimation of the inhalation risk (IR), using Equation (6):

(6) $$IR=CDI\times IUR=\frac{C_{i}\cdot EF\cdot ED\cdot ET}{AT\cdot LT}\times IUR$$

In Equation (6), CDI is the chronic daily intake (µg/m^3^) and IUR is the inhalation unit risk, obtained by the risk assessment information system [41].

About the exposure parameters (EF, ED, ET), these values are strictly correlated with the working activity of examiners. The EF (exposure frequency) is correlated with the samples analyzed by a single examiner during the working activity; ED (exposure duration) is the years of active work performed by each examiner (N_Y_); ET (exposure time) is equal to the number of sample presentations (N_PR_) during the rounds (N_R_) of analysis multiplied by the inhalation time (IT) for every presentation and the frequency of analyzed samples during the survey period (F_S_). The lifetime (LT) and averaging time (AT) parameters are equal to 365 days/year (AT) and 70 years (LT), respectively, according to the U.S. EPA definition [42].

Following this, Equation (6) could be rewritten, on the base of specific criteria for olfactometric analyses, as follows:

(7) $$IR=\frac{C_{in,i}\cdot N_{Y}\cdot F_{S}\cdot N_{PR}\cdot N_{R}\cdot IT}{AT\cdot LT}\times IUR$$

An acceptable carcinogenic risk level for workers is defined for an IR lower than 10^−5^ for mixtures [7]. Section 3.3.3 should be referred to for a more detailed discussion of the acceptability criteria considered by applying the probabilistic approach.

### 3.3. Probabilistic Approach

The application of a deterministic approach may not be able to the variability of the exposure for the particular scenario of olfactometric analyses. Therefore, the estimation of non-cancer and cancer risk using a probabilistic approach provides a solution for considering the large variability associated with the exposure of olfactometric workers. The real exposure concentrations and exposure parameters may be the input for a Monte Carlo simulation, randomizing all the variabilities present in this particular exposition scenario.

This probabilistic assessment allows the introduction of the randomness derived from the abovementioned variabilities and, thus, provides for the calculation of a prospective hazard’s likelihood while quantitatively accounting for unpredictability in the risk evaluation parameters (i.e., exposure data) [11,12,43,44,45,46,47,48].

Considering the olfactometric panelists’ scenario, the starting point of this approach is the collection of a real-scenario dataset, both in terms of exposure concentration (Cin_,i_, by overlapping chemical and odor concentration as exposed in Equation (3)), and in terms of exposure parameters (i.e., ED, EF and ET). Regarding the exposure parameters, as described in Section 2.3, in this context, the classical exposure parameters (ED, EF, and ET) have to be evaluated according to panelists’ exposure: therefore, the collected and randomized exposure raw data are N_PR_, F_S_, and N_Y_. To clarify, Table 1 describes the connection between these specific data and the classical exposure parameters (ED, EF, and ET) and the randomization of these parameters.

**Figure 4:**
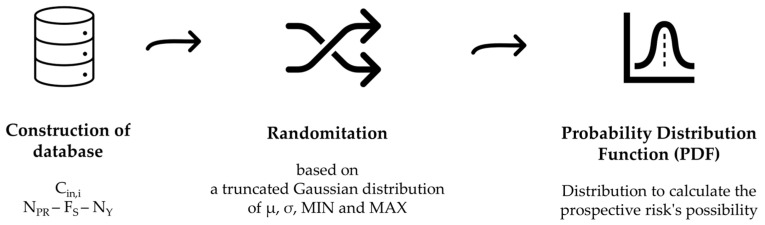
Schematization of elaboration step.

Given the availability of a real-case dataset for all these variables, it is possible to estimate descriptive statistics parameters (i.e., the mean, standard deviation, and minimum and maximum values) and consequently to simulate a distribution representative of the theoretical population of every single parameter, both for Cin_,i_ and the exposure parameters. A schematization of the workflow to apply this probabilistic approach is reported in Figure 4.

To calculate the probability distribution function (PDF), a truncated Gaussian distribution is adopted. The parameters of this distribution are the mean (μ), the standard deviation (σ), and the minimum (MIN) and maximum (MAX) values. A truncated Gaussian distribution is a variant of the normal one, with restricted values in a specific range, in this case, the MIN e MAX values observed from the parameters database. For each considered parameter (Cin_i_, N_PR_, F_S_, and N_Y_), the randomized values are used to generate the overall risk PDF.

#### 3.3.1. Non-Carcinogenic Risk—Hazard Index (HI) Evaluation

When assessing the non-carcinogenic risk [7], it must be considered that the HI approach does not contemplate exposure parameters (in terms of exposure time, frequency, and duration) other than the exposure concentration.

The non-carcinogenic risk (Equation (8)) can be statistically evaluated by the Monte Carlo randomization of the exposure concentration value, obtained as the inhalation concentration, Cin_,i_ (by Equation (3)). As mentioned, the randomization of Cin_i_ is based on a truncated Gaussian distribution of sample mean and standard deviation of each detected compound and truncated to the minimum and maximum inhalation real-case dataset values. The subscript MC denotes that the specified variable is subject to randomization according to the described Monte Carlo simulation.

(8) $$HI=\sum_{i}\frac{{\left({C_{in}}_{i}\right)}_{MC}}{{OEL}_{i}}$$

#### 3.3.2. Carcinogenic Risk—Inhalation Risk Evaluation

According to the existing guidelines and the methodology already proposed, the determination of carcinogenic risk, by calculating the IR, requires the definition of the chonic daily intake (CDI).

To evaluate the carcinogenic risk (Equation (9)), this probabilistic approach considers the selection of the exposure concentration values randomly based on the inhalation concentration, Cin_,i_ (by Equation (3)) and the exposure factors (N_PR_, F_S_, N_Y_), summarized in Table 1.

(9) $$IR=\left[{\left(\sum_{i}{C_{in}}_{i}\right)}_{MC}\cdot {IUR}_{i}\right]\cdot \frac{{{(N}_{Y})}_{MC}\cdot {\left(F_{S}\right)}_{MC}\cdot {\left(N_{PR}\right)}_{MC}\cdot N_{R}\cdot IT}{AT\cdot LT}$$

It is necessary to highlight, as described in Section 2.3. and in Table 1, that the number of rounds (N_R_), and the inhalation time (IT) do not require a randomization because, in the same laboratory condition, these values are defined. In addition, according to their definition, the lifetime (LT) and averaging time (AT) parameters are fixed, and they do not require randomization: these values are equal to 365 days/year (AT) and 70 years (LT), respectively, according to the U.S. EPA definition [42,49]. It is worth noting that this approach adds further conservativeness to the analysis: in fact, all presentations of the diluted sample are considered to occur at the confirmatory concentration, while the N_PR_-1 previous presentations occur at lower concentrations.

#### 3.3.3. Risk Distribution and Acceptability Criteria

The raw output of the presented stochastic approach is a PDF of HI and IR for non-carcinogenic and carcinogenic risk, respectively, for each considered industrial category. Thus, probabilistic assessment allows the quantitative estimation of a risk’s probability. In a deterministic approach, a risk scenario would be considered unacceptable if HI ≥ 1 and/or IR ≥ 10^−5^ [7]. This criterion is still valid, obviously, but the probability of occurrence must be introduced in this method. Therefore, it is possible to calculate the probability of an unacceptable risk, allowing for a more refined risk characterization. For example, it is recommended that compliance with the HI and IR threshold is achieved by considering the 95th percentiles of both the calculated HI and IR PDFs. In other words, to conduct the risk assessment, the non-exceedance of thresholds for HI and IR is assessed for at least 95% of the calculated exposure scenarios [11,12,50]. To evaluate the robustness of the estimated risk value (the respect of acceptability criteria for HI and IR at the 95th percentile), different statistical parameters (arithmetic mean, standard deviation, and risk values at the 25th, 50th, and 75th percentile) can also be assessed. Lastly, a sensitivity test should be carried out to determine how much the number of iterations of its parameters affects the obtained results.

The proposed approach, although based on a real-case dataset (and which, therefore, can be used to assess risk retrospectively), is also configured for a provisional approach: the calculated risk provides information on which sample types or emissions sources may have a higher probability of risk, enabling a predictive evaluation of the protection strategies that may be most effective for the safety of olfactometric employees in hypothetical future exposure scenarios. The PDF may be used to generate a cumulative distribution function (CDF), which can be easily used to cross-reference the risk threshold and the exceedance probability percentile (95th).

## 4. Probabilistic Risk Assessment: Application to Real Exposure Scenario

### 4.1. Data Collection

The probabilistic approach here constructed and proposed, was applied to a real-case exposure scenario, to estimate the risk, in terms of probability, associated with the working activity of olfactometric examiners.

To apply this approach, an intensive exposure data collection, conducted for all the parameters described in Section 2.3 and reported in Figure 3, was conducted by an annual survey of the working activity of the olfactometric panel of a university laboratory (i.e., the *Laboratorio Olfattometrico* of PoliMI, Milan, Italy).

#### 4.1.1. Dynamic Olfactometry: Collection of C_od_

During the annual survey, information about the *exposure concentration* was collected.

As previously described, the concentration inhaled by panelists during olfactometric examiners can be seen as equal to the concentration of chemicals present in the sample divided by the odor concentration (C_od_) evaluated by dynamic olfactometry. Therefore, the determination of odor concentration by dynamic olfactometry is the first step of this elaboration.

According to EN 13725:2022, C_od_ refers to the quantity of neutral air dilutions required to obtain an odorous sample to its odor detection threshold concentration. The olfactometric analyses were conducted by using the olfactometer—model TO8 by ECOMA GmbH, based on the “Yes/No” method. Panelists were selected according to the EN 13725 prescriptions. All the olfactometric measurements were conducted by involving a panel of four examiners. The odor samples to be analyzed were collected directly at odor sources in Nalophan^TM^ bags, equipped with a Teflon^TM^ inlet tube. To collect the gas directly into the sampling bag due to the depression and to avoid the measured gas from being contaminated, air samples were collected using a vacuum pump in the case of point sources. Sampling on area sources (e.g., liquid surface) was carried out using a wind tunnel system [51,52].

For the mentioned real-case dataset, n = 1035 samples, from different industrial categories, were analyzed by dynamic olfactometry. The samples’ categories and the number of analyzed samples, divided into the different industrial categories, are reported in Table S1.

#### 4.1.2. Chemical Analysis: Nature and Concentration of Pollutants Present in the Analyzed Samples

To investigate the chemical composition, during the survey, samples were chemically analyzed to determine the composition of the gaseous mix to assess the concentration of each compound. It was chosen to chemically evaluate a representative fraction, attempting to correspond with the percentage of olfactometrically analyzed samples: in particular, 25% of the total dynamic olfactometry samples were chemically analyzed (258 out of 1035 samples). The samples’ categories and the number of analyzed samples, divided into the different industrial categories, are reported in Table S1.

The chemical analysis was conducted following a previous study [53], using a gas chromatograph coupled with three detectors (a single-quadrupole mass selective detector, MS; a flame ionization detector, FID; a pulsed-flame photometric detector, PFPD). By using a calibrated pump (Markes, Air Server-xr), air samples were collected directly from the Nalophan^TM^ sampling bags utilized for the olfactometric measurements and sent to thermal desorption (Unity-xr^TM^, Markes International, Llantrisant, UK). The gas was moved, after the sampling from the bag, directly to a cold trap (‘TO-15/TO-17 Air toxics’, compatible with the simultaneous analysis of analytes from C_2/3_ to C_30/32_, Markes International, Llantrisant, UK) and maintained at −27 °C. The thermal desorption was conducted by heating the trap from −27 °C to 300 °C and the compounds were sent by hot transfer line (200 °C) into the capillary column (DB-sulfur SCD, 60 m × 0.320 mm × 4.20 μm, Agilent J&W, Folsom, CA, USA). A capillary flow technology splitter (Agilent Splitter CFT) at the end of the chromatographic column divides the gas flow following the chromatographic run into equal sections for the three detectors. The identification of the compounds was conducted by comparing the spectra obtained by GC-MS analyses with the NIST20 database (NIST/EPA/NIH Mass Spectral Library, Version 2.4 25 March 2020). Quantitative estimation of analytes was conducted by GC-FID using an external standard calibration method, using standard cylinders containing various odorous and/or volatile compounds at known concentrations (SIAD SpA, Bergamo, Italy), to obtain a molecule-specific response factor (RF). The concentration of the compounds present in the standard gas mixtures was calculated by direct calibration, using the specific calibration line constructed, while for the substances identified and not present in the standard, an appropriate family RF was used (Table S2).

The concentration values, expressed in mg/m^3^, were used for the estimation of occupational exposure risk for olfactometric examiners.

### 4.2. Evaluation of the Risk—Probabilistic Approach

#### 4.2.1. Exposure Parameters

Firstly, considering the entire working activity of panelists during the survey, some consideration about the exposure parameters should be conducted.

The exposure data with their statistical parameters collected from the yearly survey of the working activity of panelists of *Laboratorio Olfattometrico* of PoliMI are provided in Table S3. According to this survey, it is possible to highlight and confirm a high degree of variability in the activity of the single olfactometry assessor. In particular, the N_Y_ and the F_S_ are characterized by the highest variability in our dataset. In addition, the wide interval between the minimum and maximum values further confirms this variability. These preliminary observations allow evidence of the importance of the adoption of a probabilistic approach to estimate occupational risk and consider such heterogeneity.

The parameters reported in Table S3 describe the global exposure of panelists working in *Laboratorio Olfattometrico* of PoliMI: indeed, the entire exposure was considered for the evaluation of these parameters, which were then applied to the individual sample categories to study the risk associated with these categories.

#### 4.2.2. Exposure Concentration (Cin_i_)

Firstly, the exposure concentration has to be randomly assigned, according to the criteria presented in the methodology development (Section 3).

**Figure 5:**
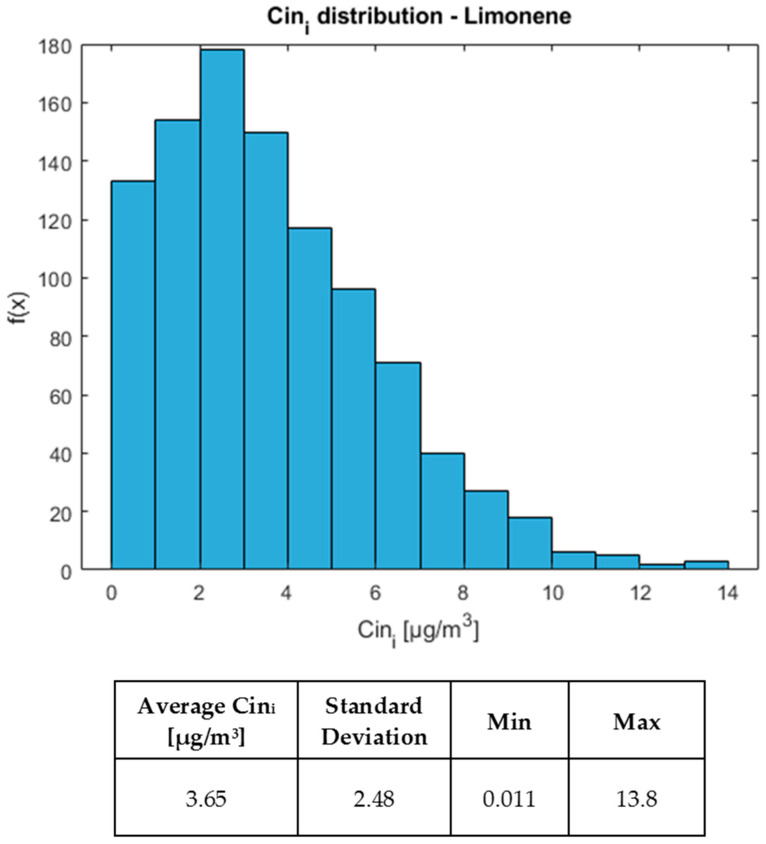
Distribution of inhalation concentration (Cin_i_) and statistical parameters for limonene in *biomass* samples.

An example of exposure concentration distribution, Cin_i_, is reported below. Figure 5 shows the graphical distribution and statistical parameters for the 1000 iterations of limonene concentrations in *biomass* samples, expressed in µg/m^3^.

Based on the reported data (Figure 5), some considerations could be drawn: the mean is relatively low, considering the minimum–maximum interval, which may suggest that the majority of the data tend to be concentrated towards the lower tail. In addition, the standard deviation is also restrained: this indicates a relatively limited dispersion of data around the average. Lastly, the difference between the minimum and maximum values is relevant (13.79 µg/m^3^), suggesting that the distribution may cover a wide range of values, although most of the data is concentrated towards the tail at low values. This single-case behaviour, here presented for the sake of example for limonene, accurately depicts the results for most of the identified molecules.

#### 4.2.3. Non-Carcinogenic Risk—Hazard Index (HI)

**Figure 6:**
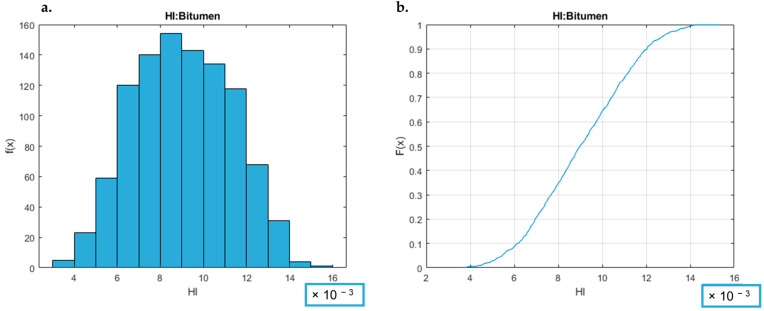
Elaboration of non-carcinogenic risk for the bitumen category. (**a**) shows the HI PDF: on the x-axis, the observed HI values are represented, and on the y-axis, the occurrence is shown. The area under the curve indicates the probability that the HI falls within that range. (**b**) shows HI CDF: on the x-axis, the observed HI values are represented, and on the y-axis, the cumulative probability is shown. The curve increases as the HI value increases, reaching 1 when all possible values are covered.

By way of example, the HI graphs (PDF and CDF) for the *bitumen* samples category are reported in Figure 6. The CDF and PDF graphs of all the investigated sample categories are reported in Supplementary Materials.

As shown in Figure 6 (and in Figures S1–S10 of Supplementary Materials), all the analyzed categories respect the risk acceptability criteria (HI < 1). This analysis of the PDF and CDF graphs (Figure 6 and Figures S1–S10 of Supplementary Materials) indicates that the non-carcinogenic risk is negligible for all the considered plant categories.

In Table 2, the statistical parameters (the arithmetic mean, standard deviation, and sample HI values at the 25th, 50th, 75th, and 95th percentile), assessed to evaluate the robustness of the risk estimation, are reported for all the investigated 13 sample categories.

As preliminarily observed from CDF and PDF graphs, the data reported in Table 2 also highlight that the non-carcinogenic risk estimations, for all the considered sample categories, are lower than the acceptability criteria (HI < 1) at the considered frequency, i.e., the 95th percentile. Evaluating the single samples classes, it is possible to observe that the highest HI values, although always about an order of magnitude lower than the acceptability criterion, are observed for the petrochemical categories, followed by the *hydrocarbon tanks* and *refinery* categories.

In addition, by observing the PDF and CDF graphs (Figures S1–S10 of Supplementary Materials), it can be deduced that all the maximum values (i.e., the HI estimations for the worst-case scenarios of each category of samples, 100th percentile), even if they are individual stochastic values, are far lower than the acceptability criteria. Therefore, overall, the probability of observing a situation of risk (HI > 1), estimated on the basis of the observations obtained from the laboratory case study, can be considered negligible.

From these observations, it is possible to affirm that the probability of observing a relevant non-carcinogenic risk associated with the panelists’ activity, based on the considered annual survey of the considered laboratory, can be considered negligible.

#### 4.2.4. Carcinogenic Risk—Inhalation Risk (IR)

**Figure 7:**
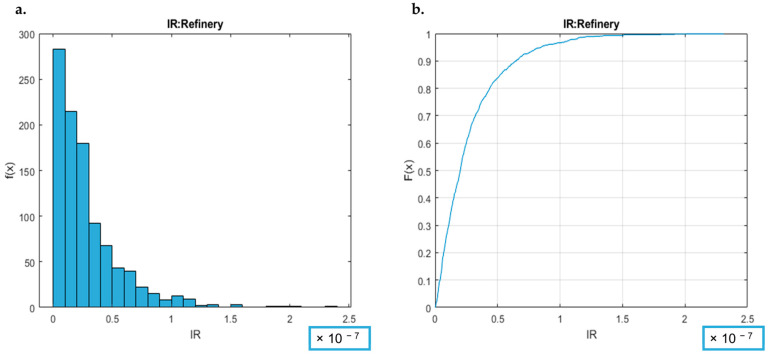
Elaboration of non-carcinogenic risk for the *refinery* category. (**a**) shows IR: on the *x*-axis, the observed IR values are represented, and on the *y*-axis, the probability density is shown. The area under the curve indicates the probability that the IR falls within that range. (**b**) shows IR CDF: on the *x*-axis, the observed IR values are represented, and on the *y*-axis, the cumulative probability is shown. The curve increases as the IR value increases, reaching 1 when all possible values are covered.

For analyzing the carcinogenic risk, by way of example, the IR’s CDF and PDF graphs for the *refinery* samples category are reported in Figure 7.

Figure 7 (and Figures S11–S18 of Supplementary Materials) report the distribution plots of IR for the investigated categories, while in Table 3, the statistical parameters (arithmetic mean, standard deviation, and risk values at the 25th, 50th, 75th, and 95th percentiles), assessed to evaluate the robustness of the risk estimation, are reported for the 13 sample categories. The PDF and CDF graphs (Figure 7 and Figures S11–S18 of Supplementary Materials) indicate that the non-carcinogenic risk is negligible for the plant categories under consideration.

As preliminarily observed from the CDF and PDF graphs, Table 3 shows that the carcinogenic risk for all the considered sample categories are lower than the acceptability criterion (IR < 10^−5^): at the 95th percentile in particular, the value of IR, derived from the Monte Carlo elaboration, is always lower than 10^−5^ at least for two orders of magnitude.

Similarly as described for the non-cancer risk, the data in Table 3 and the CDF graphs (Figures S11–S14) show not only that the acceptability criterion of IR < 10^−5^ is respected at the considered frequency (95th percentile), but also that the maximum values (i.e., the HI estimations for the worst case scenarios of each category of samples, 100th percentile) are always orders of magnitude lower than this acceptability limit. As already mentioned for non-carcinogenic risk (Section 4.2.3.), focusing on the PDF and CDF graphs (Figures S11–S18), it is possible to observe that the probability of observing a situation of risk (IR > 10^−5^), estimated based on the observations of the present laboratory, can be considered negligible.

For two categories (*WWTP* and *Biofuel*), the distribution of carcinogenic risk could not be calculated due to the absence of carcinogenic compounds in the analyzed samples of these categories.

It is, thus, possible to affirm that the probability of observing a relevant carcinogenic risk, associated with the panelists’ activity, based on the considered annual survey of the considered laboratory, can be considered negligible, and no risk situations are identified.

### 4.3. Sensitivity Analysis

**Figure 8:**
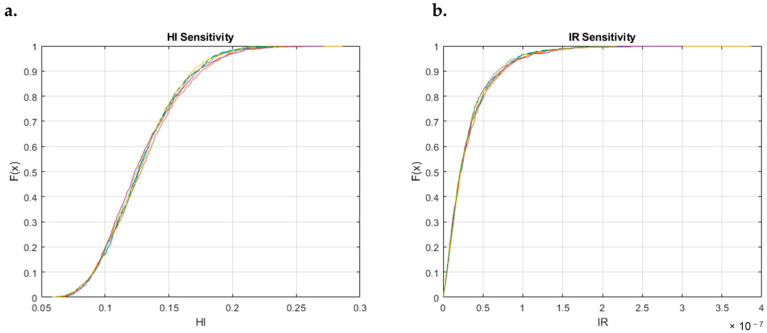
Sensibility test for HI (**a**) and IR (**b**) for the *petrochemical (cracking)* category: CDF of 5 interactions.

To assess the robustness of the approach conducted and to determine how much the number of iterations of parameters affects the obtained results, a sensitivity test was conducted for the non-carcinogenic and the carcinogenic risk. The test was conducted for all categories. For the sake of brevity, only the outcome for the *petrochemical (cracking)* category is reported. The results of five iterations are reported, both for HI and IR, in Figure 8.

The number of iterations for parameter randomization results to be sufficient to ensure the robustness and consistency of the system: no significant differences among iterations are detected (overlap between the 5 CDFs), implying that the randomization process is sufficiently resilient to be unaffected by single-run randomization.

## 5. Strengths and Limitations of the Approach

Although the application of a probabilistic approach, as intended in this case study, is a very convenient tool to investigate the exposure risk for panelists, due to their great exposure variability, some assumptions are adopted in this study.

The first assumption conducted is related to the selection of occupational exposure Limits (OEL). In this study to evaluate the HI of the mixture, the OELs adopted in the elaboration are selected according to the hierarchical order defined in previous studies [7,35]. In general, the threshold value for risk characterization must be chosen with considerable care, especially when limit values are being amended (considering that threshold values are often changed downwards). From these observations, it is possible to affirm that difficulties may arise in the selection of the most appropriate OEL values, it remains a very binding criterion and must be decided with caution.

It is also worth noting that HI is a simplified approach that does not consider chemical interactions and toxicokinetic or toxicodynamic differences. However, this approach remains one of the more common in the literature about occupational risk for olfactometric panelists [7,22,23,24,31,35] and is the most simple, flexible, robust, and conservative approach, particularly if applied in this context.

Despite these drawbacks, the study has several advantages because it sheds light on the risk assessment process for panelists exposed to odor samples in dynamic olfactometry, a unique occupational exposure scenario marked by the impossibility of applying the traditional hierarchy of risk management and mitigation measures (i.e., elimination/replacement of the risk agent, confinement of the risk agent, use of collective protection devices, and the use of personal protective equipment). The application of the probabilistic approach allows the estimation of the occupational risk associated with the working activities of examiners involved in olfactometric tests, considering all the variabilities among the specific panelists’ activities. The key advantage of analyzing risk probability is the capacity to assess the overall exposure scenario, considering the total number of studied olfactometric samples even though only a portion of them have been chemically characterized.

In addition, the study was conducted using a further largely conservative approach. Firstly, according to the mentioned hierarchical order, an OEL of 8 h was selected for those compounds for which a short-term exposure value is absent, even though the panelists’ exposure is substantially shorter than 40 h/week: OEL 8 h values are, according to the higher exposure time, lower than the corresponding short-term values for the same substance. In addition, the use of a multiplication factor k equal to 10 for the determination of the inhalation concentration (Cin,_i_) is an additional conservative safety parameter: this is the maximum deviation value that can be obtained from the different panels. Even though this condition is only possible, although very rare in laboratory experience, it was considered to be achieved for every single measurement. Furthermore, considering Cin_i_, a further largely conservative hypothesis was considered: even though the real inhaled concentrations are far lower than the final one, due to the dilution mechanism of the olfactometer, by a factor 2^n^, it was considered that for all the presentations the panels inhale the highest concentration of the whole sample analysis.

Finally, to put all categories on an equal level and to maintain a conservative approach, it was considered that all samples analyzed by the panel belonged to each individual category and, for each, the potential risk scenario was calculated.

Therefore, the results obtained from the annual survey of examiners working in *Laboratorio Olfattometrico* of PoliMi can be used as an indication of the risk to which these workers are exposed (also ensuring the use of a conservative approach), especially considering the large number of experimental data used within the study. Therefore, a similar approach can be extrapolated from this study and applied to other scenarios of workers involved in olfactometric analysis.

## 6. Conclusions

This study aims to propose and apply a probabilistic methodology to estimate the occupational risk for examiners involved in olfactometric analysis. The principal benefit of estimating the probability of risk incidence is the ability to evaluate the overall exposure scenario, even though only a portion of the olfactometric samples studied have been chemically characterized. The approach suggested in this article is based on a Monte Carlo stochastic evaluation of both cancer-causing and non-cancerous risk and the obtainment of a probability distribution of the risk: this overall risk scenario could be compared with the acceptability criteria defined for the risk parameters (HI and IR).

This model aims to overcome the problem correlated with previous risk assessment studies applied to dynamic olfactometry panels (i.e., those based on a deterministic approach) and to suggest a strategy to characterize, from a comprehensive perspective, the occupational risk connected with this sensorial analysis. From the point of view of olfactometric laboratories, and related workers, assessing a priori the probability of the risk associated with the determination of odor concentration, for different types of industrial emissions, is crucial for the development of this activity while protecting the health of the personnel involved, as required by EN 13725.

From the application of this probabilistic approach to a real-case exposure scenario, PDF and CDF graphs were constructed to evaluate the non-carcinogenic and carcinogenic risk, and an evaluation of the risk indicator values at the 95th percentile of the generated stochastic sample was conducted, to assess if they respect the acceptability criteria considered (HI < 1 and IR < 10^−5^). From the results obtained in this study, it is possible to affirm that, despite the large number of conservative hypotheses, HI and IR have been shown to always be lower than the defined acceptability criteria. From this evaluation, it is possible to conclude that the probability of observing non-carcinogenic and carcinogenic risk, associated with the activity of panelists, based on the considered annual survey, can be considered negligible.

Some assumptions and limitations must be considered when interpreting this study’s results: the common criterion has been always the cautious approach.

However, despite the limitations and the difficulties connected with the estimation of certain parameters, the application of a probabilistic approach appears to be a very useful and comprehensive tool for assessing the risk associated with this activity and for evaluating, assuming the same conditions of analysis, this overall particular exposure scenario.

## Figures and Tables

**Table 1:** Relation between exposure parameters and exposure factor for olfactometric risk evaluation.

Exposure Parameters	How to Calculate Them by Panel Exposure	Can It Be Randomized?
Exposure time ET [hours/days]	By multiplying the inhalation time (IT) and number of presentations (N_PR_) during the different rounds (N_R)_: ET =N_PR_ · N_R_ · IT.	ET can be estimated by randomizing only N_PR_ (IT and N_R_ are fixed and pre-determined according to the analysis conditions).
Exposure frequency EF [days/year]	Estimating the number of analyzed samples in the survey period (F_S_).	EF can be randomized according to the specific examiner activity.
Exposure duration ED [years]	By evaluating the years of working activity for each examiner (N_Y_).	ED can be randomized according to the specific examiner activity.

**Table 2:** Statistical summary of the HI dataset, including arithmetic mean, standard deviation, and sample HI values at the 25th, 50th, 75th, and 95th percentiles.

Samples Category	Mean	Dev. Std.	25th Percentile	50th Percentile	75th Percentile	95th Percentile
Refinery	4.57 × 10^−2^	1.52 × 10^−2^	3.47 × 10^−2^	4.35 × 10^−2^	5.54 × 10^−2^	7.39 × 10^−2^
Petrochemical (cracking)	1.29 × 10^−1^	3.26 × 10^−2^	1.05× 10^−1^	1.25 × 10^−1^	1.48 × 10^−1^	1.88 × 10^−1^
Petrochemical (other)	1.06 × 10^−1^	3.59 × 10^−2^	7.79 × 10^−2^	1.05 × 10^−1^	1.30 × 10^−1^	1.66 × 10^−1^
Hydrocarbons tanks	6.99 × 10^−2^	1.98 × 10^−2^	5.59 × 10^−2^	6.91 × 10^−2^	8.15 × 10^−2^	1.06 × 10^−1^
WWTP	1.54 × 10^−2^	6.41 × 10^−3^	1.04 × 10^−2^	1.47 × 10^−2^	1.96 × 10^−2^	2.72 × 10^−2^
MSW	2.32 × 10^−3^	3.38 × 10^−4^	2.09 × 10^−3^	2.32 × 10^−3^	2.56 × 10^−3^	2.87 × 10^−3^
Biomass	2.40 × 10^−2^	9.97 × 10^−3^	1.61 × 10^−2^	2.29 × 10^−2^	3.05 × 10^−2^	4.15 × 10^−2^
Biofuel	1.54 × 10^−3^	7.97 × 10^−4^	9.17 × 10^−4^	1.53 × 10^−3^	2.14 × 10^−3^	2.91 × 10^−3^
Foundry	4.33 × 10^−3^	1.30 × 10^−3^	3.34 × 10^−3^	4.28 × 10^−3^	5.26 × 10^−3^	6.59 × 10^−3^
Bitumen	9.02 × 10^−3^	2.27 × 10^−3^	7.26 × 10^−3^	9.01 × 10^−3^	1.07 × 10^−2^	1.28 × 10^−2^
Industrial WWTP	4.16 × 10^−4^	6.62× 10^−5^	3.73 × 10^−4^	4.16× 10^−4^	4.62 × 10^−4^	5.25 × 10^−4^

**Table 3:** Statistical summary of the IR dataset, including arithmetic mean, standard deviation, and sample IR values at the 25th, 50th, 75th, and 95th percentiles.

Samples Category	Mean	Dev. Std.	25th Percentile	50th Percentile	75th Percentile	95th Percentile
Refinery	3.07 × 10^−8^	2.92 × 10^−8^	1.08 × 10^−8^	2.16 × 10^−8^	4.13 × 10^−8^	8.92 × 10^−8^
Petrochemical (cracking)	3.18 × 10^−8^	3.06 × 10^−8^	1.14 × 10^−8^	2.22 × 10^−8^	4.20 × 10^−8^	9.29 × 10^−8^
Petrochemical (other)	9.80 × 10^−8^	9.00 × 10^−8^	3.51 × 10^−8^	7.17 × 10^−8^	1.35 × 10^−7^	2.61 × 10^−7^
Hydrocarbons tanks	1.17× 10^−8^	1.21 × 10^−8^	3.64 × 10^−9^	8.05 × 10^−9^	1.53 × 10^−8^	3.37 × 10^−8^
MSW	1.39 × 10^−10^	1.41 × 10^−10^	4.23 × 10^−11^	9.30 × 10^−11^	1.89 × 10^−10^	4.15 × 10^−10^
Biomass	4.82 × 10^−9^	4.40 × 10^−9^	1.76 × 10^−9^	3.55 × 10^−9^	6.51 × 10^−9^	1.34 × 10^−8^
Foundry	1.10× 10^−9^	9.82 × 10^−10^	4.04 × 10^−10^	8.46 × 10^−10^	1.52 × 10^−9^	2.89 × 10^−9^
Bitumen	1.15 × 10^−10^	1.11 × 10^−10^	3.75 × 10^−11^	8.13 × 10^−11^	1.61 × 10^−10^	3.20 × 10^−10^
Industrial WWTP	9.96 × 10^−12^	8.77 × 10^−12^	3.73 × 10^−12^	7.27 × 10^−12^	1.33 × 10^−11^	2.75 × 10^−11^

## Data Availability

The data that has been used is confidential.

## Supplementary Materials

The following supporting information can be downloaded at: https://www.mdpi.com/article/10.3390/toxics12110784/s1, Table S1: Number of samples analyzed by dynamic olfactometry and chemical analysis classified into industrial categories; Table S2: Reference compounds used for quantification by GC-FID analysis; Table S3: Experimental dataset of exposure parameters. Std. Dev.: standard deviation; Min: minimum value observed; Max: maximum value observed; Figure S1: CDF of non-carcinogenic risk. Samples categories: *refinery* and *petrochemical (cracking)*; Figure S2: CDF of non-carcinogenic risk. Samples categories: *petrochemical (other)* and *hydrocarbon tanks*; Figure S3: CDF of non-carcinogenic risk. Samples categories: *Civil WWTP* and *MSW*; Figure S4: CDF of non-carcinogenic risk. Samples categories: *biomass* and *biofuel*; Figure S5: CDF of non-carcinogenic risk. Sample# categories: *foundry, bitumen*, and *industrial WWTP*; Figure S6: PDF of HI. Samples categories: *refinery* and *petrochemical (cracking)*; Figure S7: PDF of HI. Samples categories: *petrochemical (other)* and *hydrocarbon tanks*; Figure S8: PDF of HI. Samples categories: *civil WWTP* and *MSW*; Figure S9: PDF of HI. Sample categories: *biomass* and *biofuel*; Figure S10: PDF of HI. Samples categories: *foundry, bitumen*, and *industrial WWTP*; Figure S11: CDF of carcinogenic risk. Sample categories: *refinery* and *petrochemical (cracking)*; Figure S12: CDF of carcinogenic risk. Sample categories: *petrochemical (other)* and *hydrocarbon tanks*; Figure S13: CDF of carcinogenic risk. Sample categories: *MSW* and *biomass*; Figure S14: PDF of IR. Sample categories: *foundry, bitumen*, and *industrial WWTP*; Figure S15: PDF of IR. Sample categories: *refinery* and *petrochemical (cracking)*; Figure S16: PDF of IR. Samples categories: *petrochemical (other)* and *hydrocarbon tanks*; Figure S17: PDF of IR. Samples categories: *MSW* and *biomass*; Figure S18: PDF of IR. Samples categories: *foundry, bitumen*, and *industrial WWTP*.

## List of Abbreviations

AT:	Averaging time
CDI:	Chronic daily intake
C_i_:	Compound concentration
C_in,i_:	Inhalation concentration
C_od_:	Odor concentration
CDF:	Cumulative distribution function
COPs:	Chemicals of potential concern
ED:	Exposure duration
EF:	Exposure frequency
ET:	Exposure time
GC-MS:	Gas chromatography coupled with mass spectrometry
HI:	Hazard index
HQ:	Hazard quotient
IR:	Inhalation risk
IT:	Inhalation time
IUR:	Inhalation unit risk
LT:	Lifetime
MC:	Monte Carlo
MDV:	Minimum dilution value
N_PR_:	Number of presentations
N_R_:	Number of rounds
F_S_:	Frequency of analyzed samples, evaluated as number of analyzed samples oven the survey period
N_Y_:	Number of working years
NIOSH:	National Institute for Occupational Safety and Health
OEL:	Occupational exposure limit
OHS:	Occupational health and safety
PDF:	Probability distribution function
PT:	Presentation time
VOCs:	Volatile organic compounds
Z_ITE_:	Individual threshold estimate, expressed as a dilution factor (definition from EN 13725:2022)
${\bar{Z}}_{\mathrm{ITE}}$:	Geometric mean of Z_ITE_ of all panel members in one measurement (definition from EN 13725:2022)
ΔZ:	Retrospective panel screening parameter (definition from EN 13725:2022)
µ:	Mean value
σ:	Standard deviation
MIN:	Minimum value
MAX:	Maximum value
