# Supplementary material for: Probabilistic Approach for Assessing the Occupational Risk of Olfactometric Examiners: Methodology Description and Application to Real Exposure Scenario
Source: Toxics. 2024 Oct 29;12(11):784. doi: 10.3390/toxics12110784 (PMC11598029; doi:10.3390/toxics12110784)
Supplement: Supplementary file 1 [file toxics-12-00784-s001.zip › toxics-3203155-supplementary.pdf]

SUPPLEMENTARY MATERIALS

Probabilistic Approach for Assessing the Occupational Risk of Olfactometric Examiners: Methodology Description and Application to Real Exposure Scenario

Elisa Polvara [1], Andrea Spinazzè [2], Marzio Invernizzi [1,*], Andrea Cattaneo [2], Domenico Maria Cavallo [2] and Selena Sironi [1]

[1] Politecnico di Milano, Department of Chemistry, Materials and Chemical Engineering "Giulio Natta",
Piazza Leonardo da Vinci 32, 20133 Milano, Italy; elisa.polvara@polimi.it (E.P.); selena.sironi@polimi.it (S.S.)

[2] Department of Science and High Technology DiSAT, Università degli Studi dell'Insubria, Via Valleggio 11, 22100 Como, Italy; andrea.spinazze@uninsubria.it (A.S.); andrea.cattaneo@uninsubria.it (A.C.); domenico.cavallo@uninsubria.it (D.M.C.)

* Correspondence: marzio.invernizzi@polimi.it

Table S1. Number of samples analyzed by dynamic olfactometry and chemical analysis classified into industrial categories.

Category	Dynamic olfactometry		Chemical analysis	
	**N° of samples**	**% distribution**	**N° of samples**	**% distribution**
Refinery	203	19.6%	44	17.1%
Petrochemical (cracking)	48	4.6%	48	18.6%
Petrochemical (other)	25	2.4%	16	6.2%
Hydrocarbons tanks	100	9.7%	29	11.2%
Civil wastewater treatment plant (WWTP)	172	16.6%	17	6.6%
Municipal solid waste (MSW)	180	17.4%	11	4.3%
Biomass	221	21.4%	66	25.6%
Biofuel	15	1.4%	3	1.2%
Foundry	6	0.6%	6	2.3%
Bitumen	50	4.8%	15	5.8%
Industrial WWTP	15	1.4%	3	1.2%
Total	1035	100%	258	100%

Table S2. Reference compounds used for quantification by GC-FID analysis.

Family of VOCs	Representative compound
Alkanes C2-C6	n-Hexane
Alkanes C7-C20	Dodecane
Cycloalkanes	Cyclohexane
Alkenes, alkynes, and dienes	Propylene
Aromatics	Benzene
Alcohols, ethers, and carboxylic acids	Isobutanol
Terpenes	Limonene (R)+
Aldehydes and ketones	Acetone
Furans	Toluene
Esters	Ethyl acetate
Sulphur	Methyl mercaptan
Halogenates	Trichloromethane
Unknown	Toluene

Table S3. Experimental dataset of exposure parameters.

Parameter	Average	Std. Dev.	Min	Max	Median	Dataset
N_Y [year]	2.80	1.99	1	9	3	51
F_S [samples/year]	99	104	1	420	60	51
N_{PR} [presentations/sample]	4.8	0.9	3	9	5	1035
N_R [round/sample]	3					
IT [s/presentation]	2.2					

***Std. Dev.**: standard deviation; **Min**: minimum value observed; **Max**: maximum value observed.*

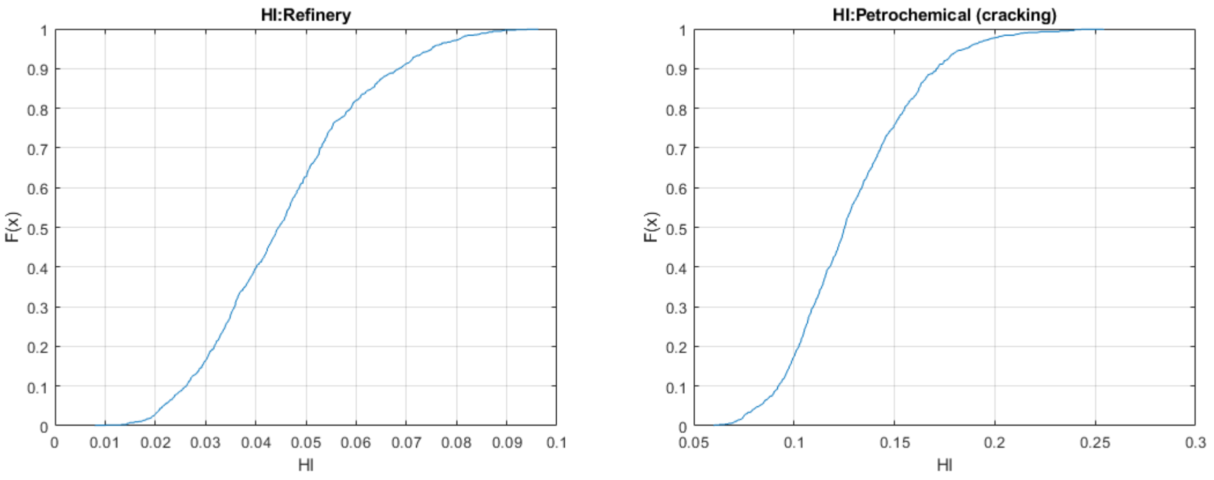

Figure S1. CDF of non-carcinogenic risk. Sample categories: *refinery* and *petrochemical (cracking)*.

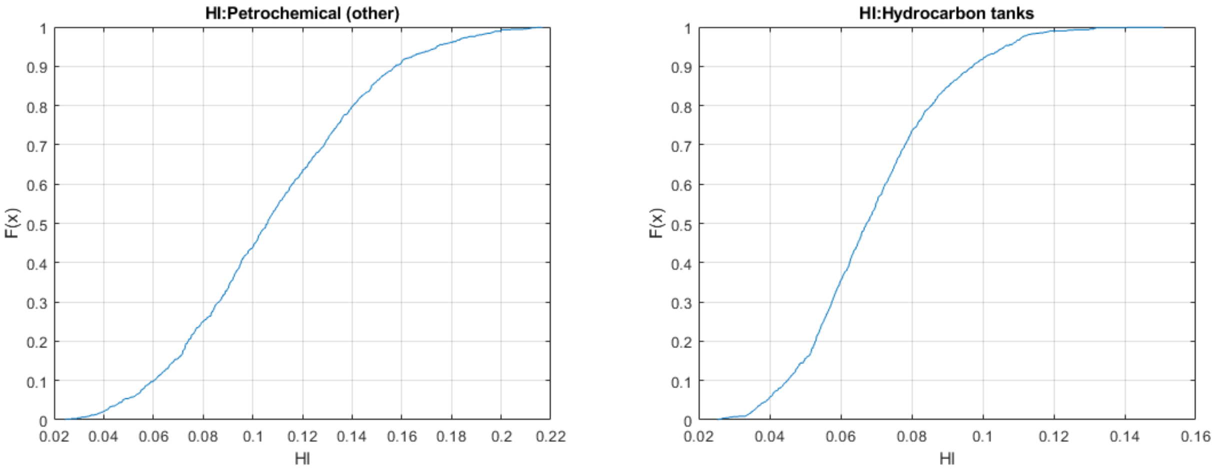

Figure S2. CDF of non-carcinogenic risk. Sample categories: *petrochemical (other)* and *hydrocarbon tanks*.

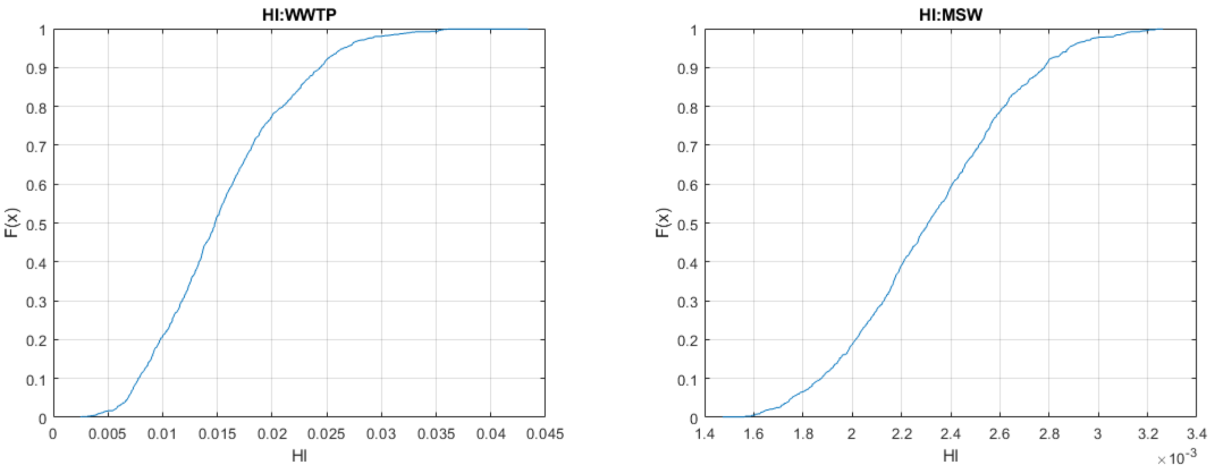

Figure S3. CDF of non-carcinogenic risk. Sample categories: *civil WWTP* and *MSW*.

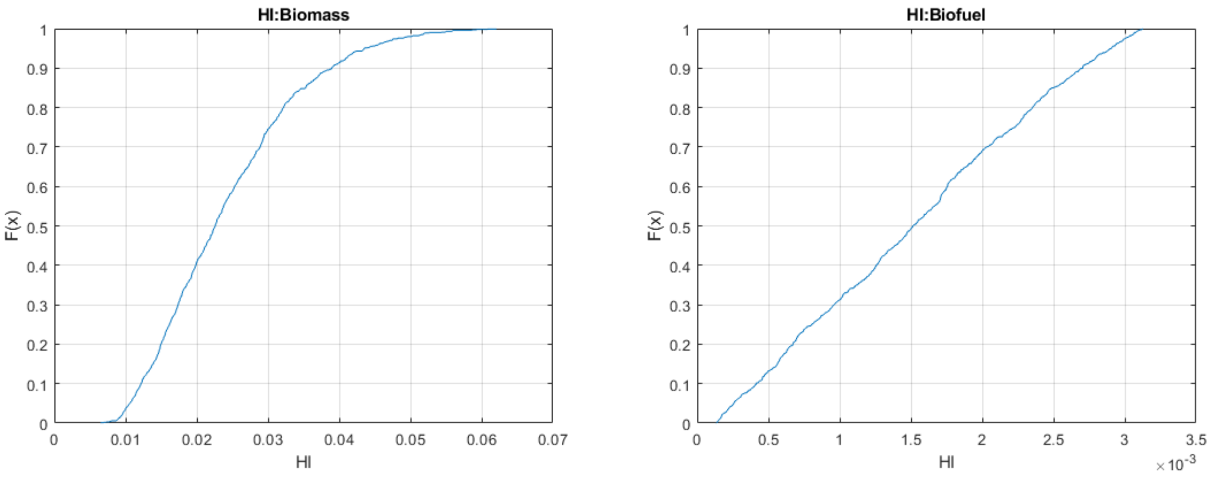

Figure S4. CDF of non-carcinogenic risk. Sample categories: *biomass* and *biofuel*.

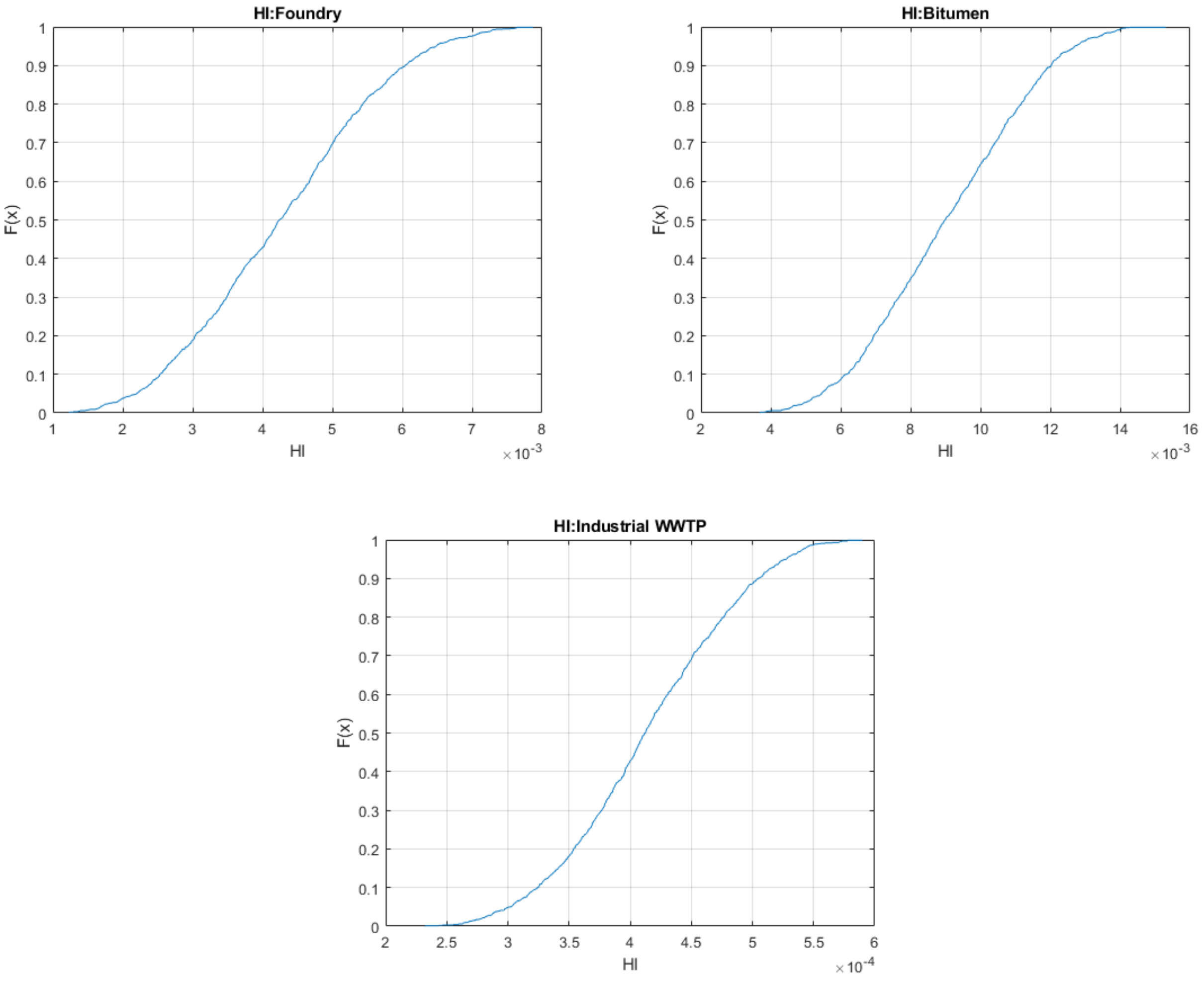

Figure S5. CDF of non-carcinogenic risk. Sample categories: *foundry*, *bitumen*, and *industrial WWTP*.

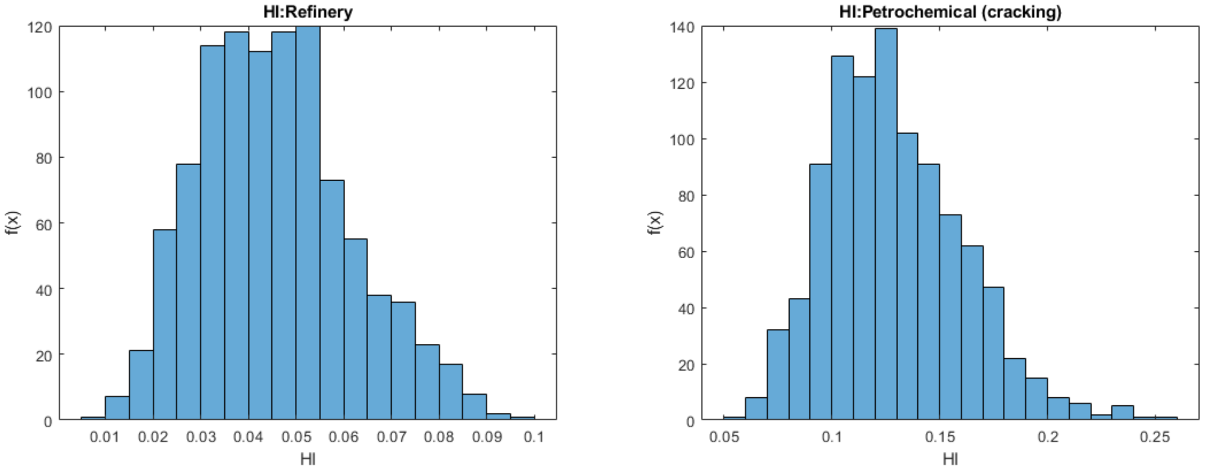

Figure S6. PDF of HI. Sample categories: *refinery* and *petrochemical (cracking).*

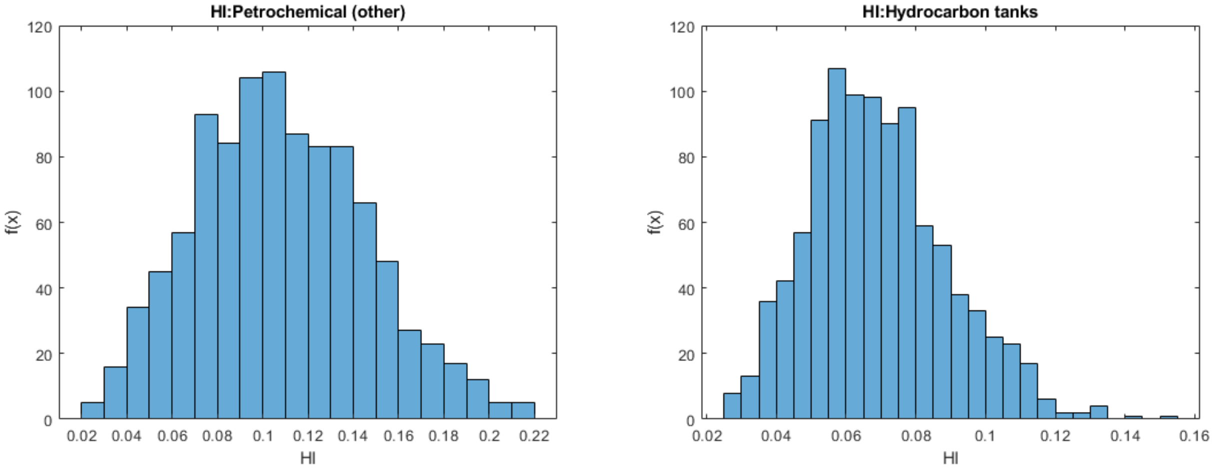

Figure S7. PDF of HI. Sample categories: *petrochemical (other)* and *hydrocarbon tanks.*

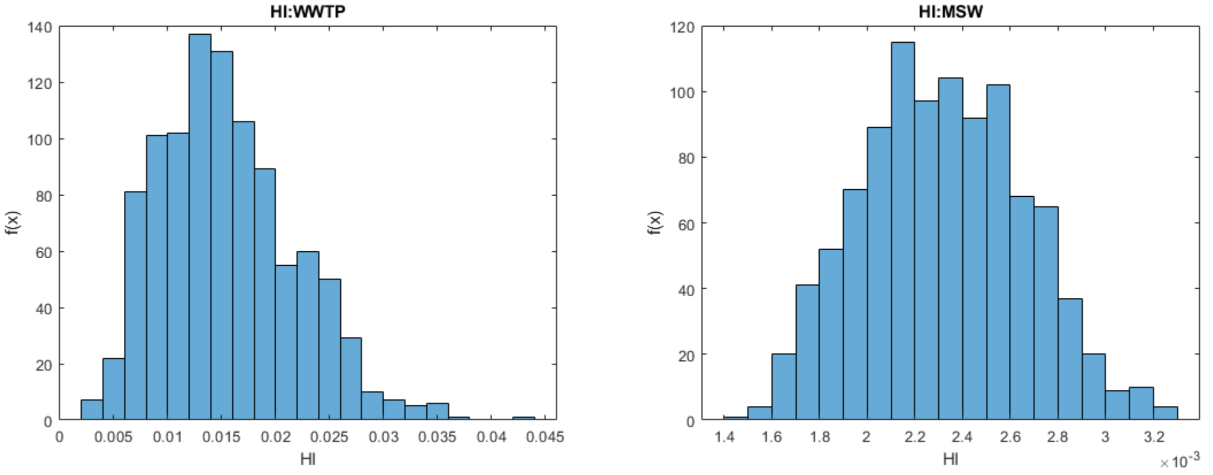

Figure S8. PDF of HI. Sample categories: *civil WWTP* and *MSW.*

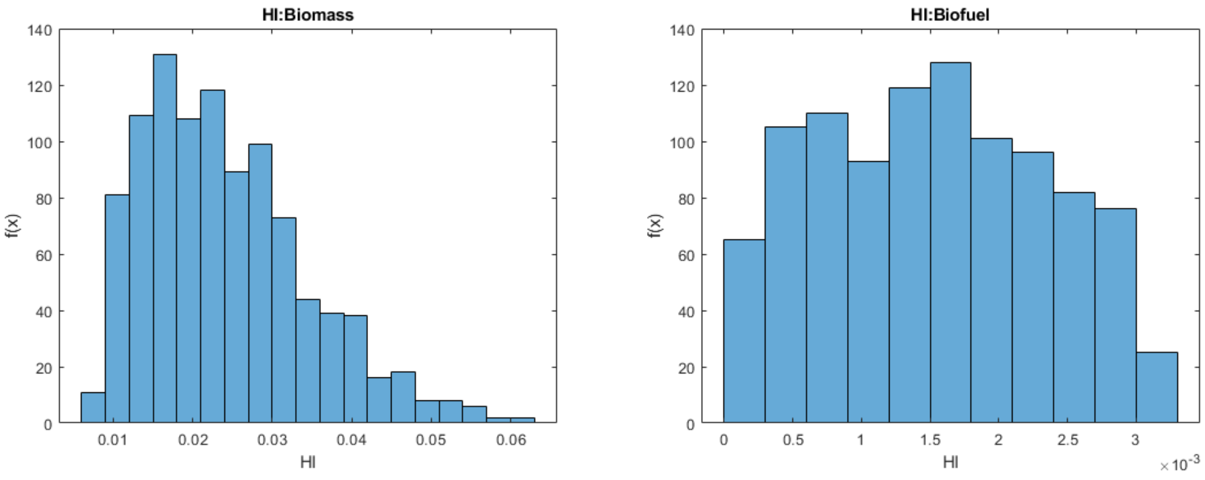

Figure S9. PDF of HI. Sample categories: *biomass* and *biofuel*.

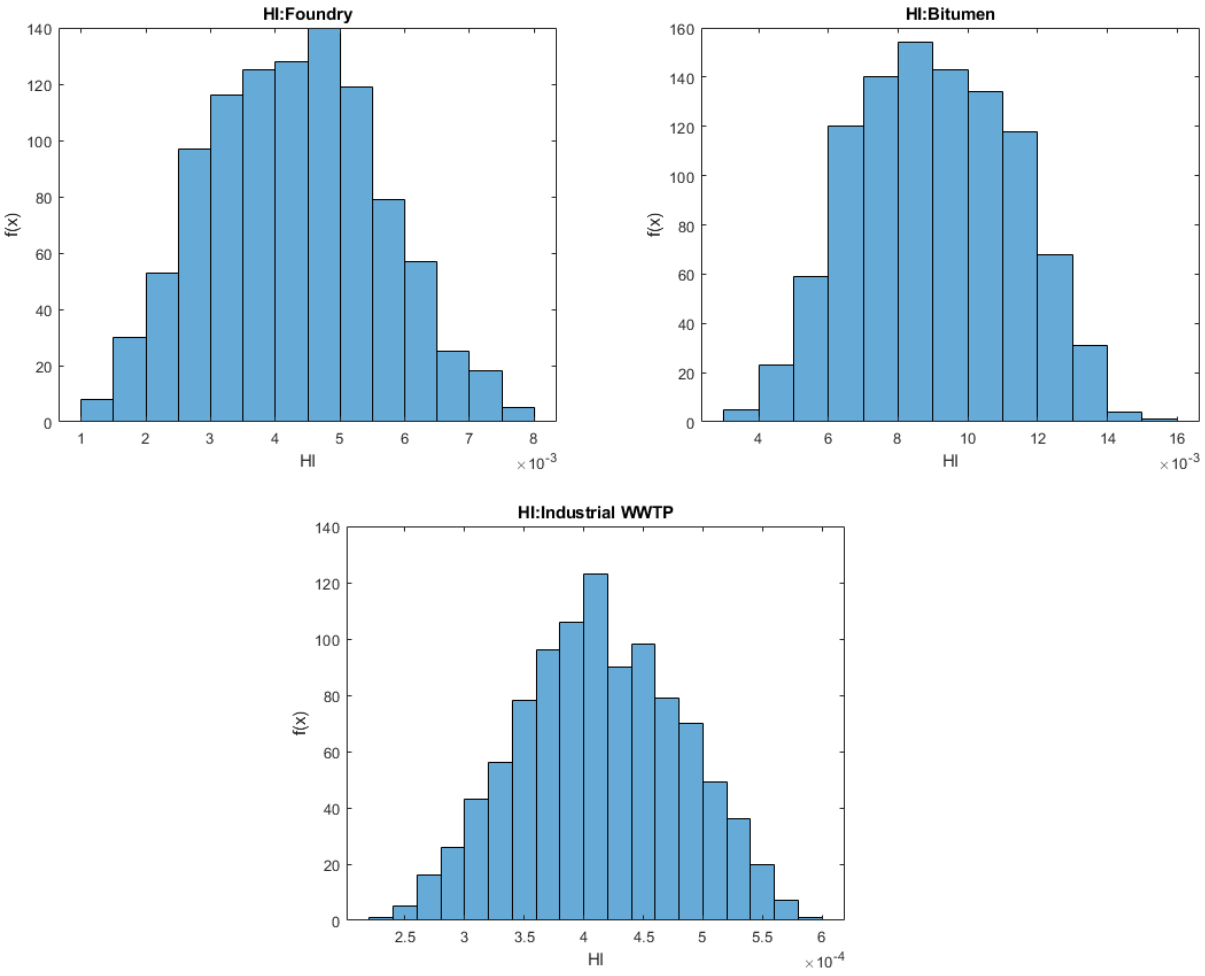

Figure S10. PDF of HI. Sample categories: *foundry, bitumen,* and *industrial WWTP*.

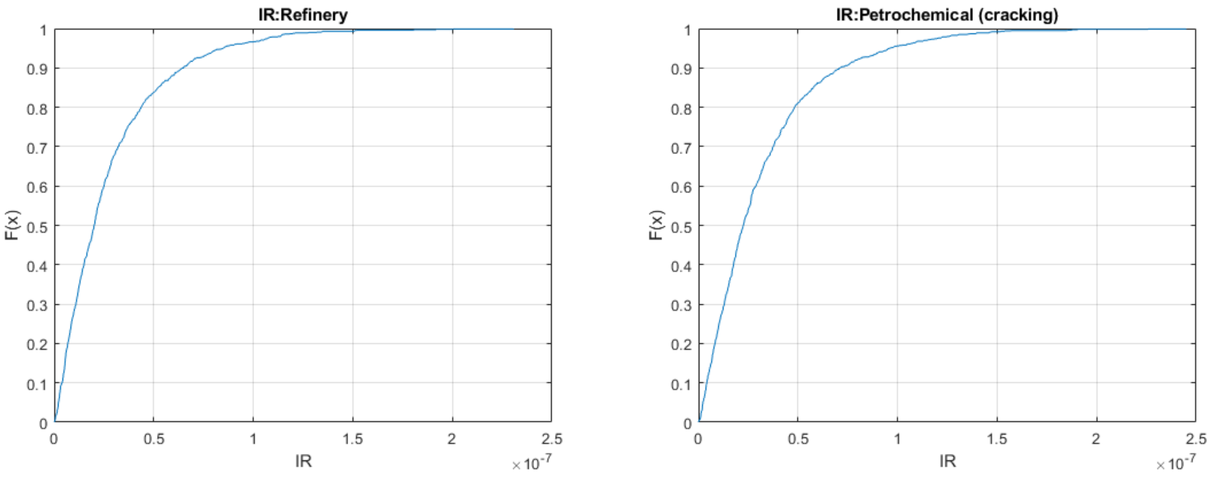

Figure S11. CDF of carcinogenic risk. Sample categories: *refinery* and *petrochemical (cracking)*.

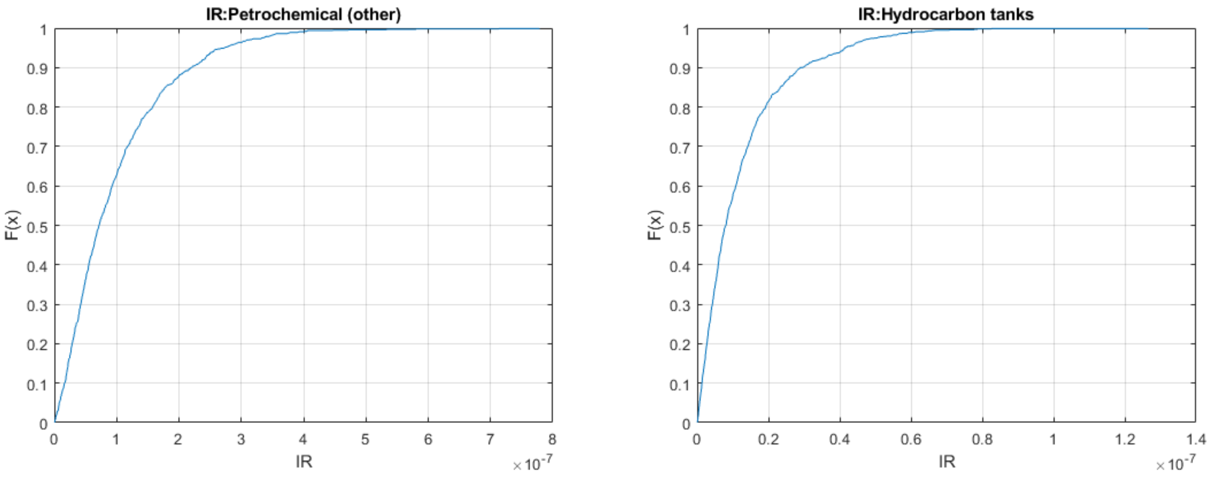

Figure S12. CDF of carcinogenic risk. Sample categories: *petrochemical (other)* and *hydrocarbon tanks*.

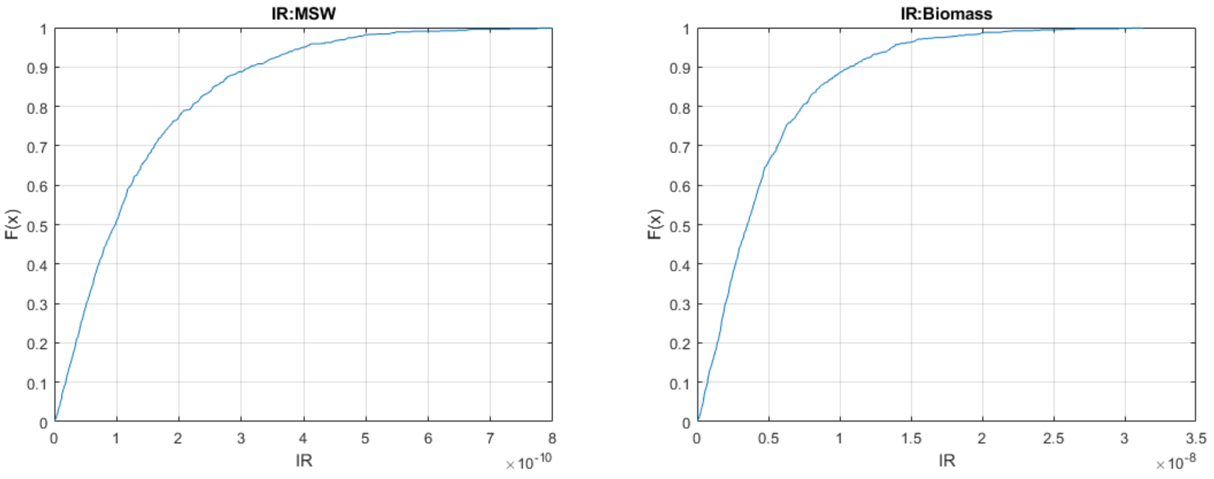

Figure S13. CDF of carcinogenic risk. Sample categories: *MSW* and *biomass*.

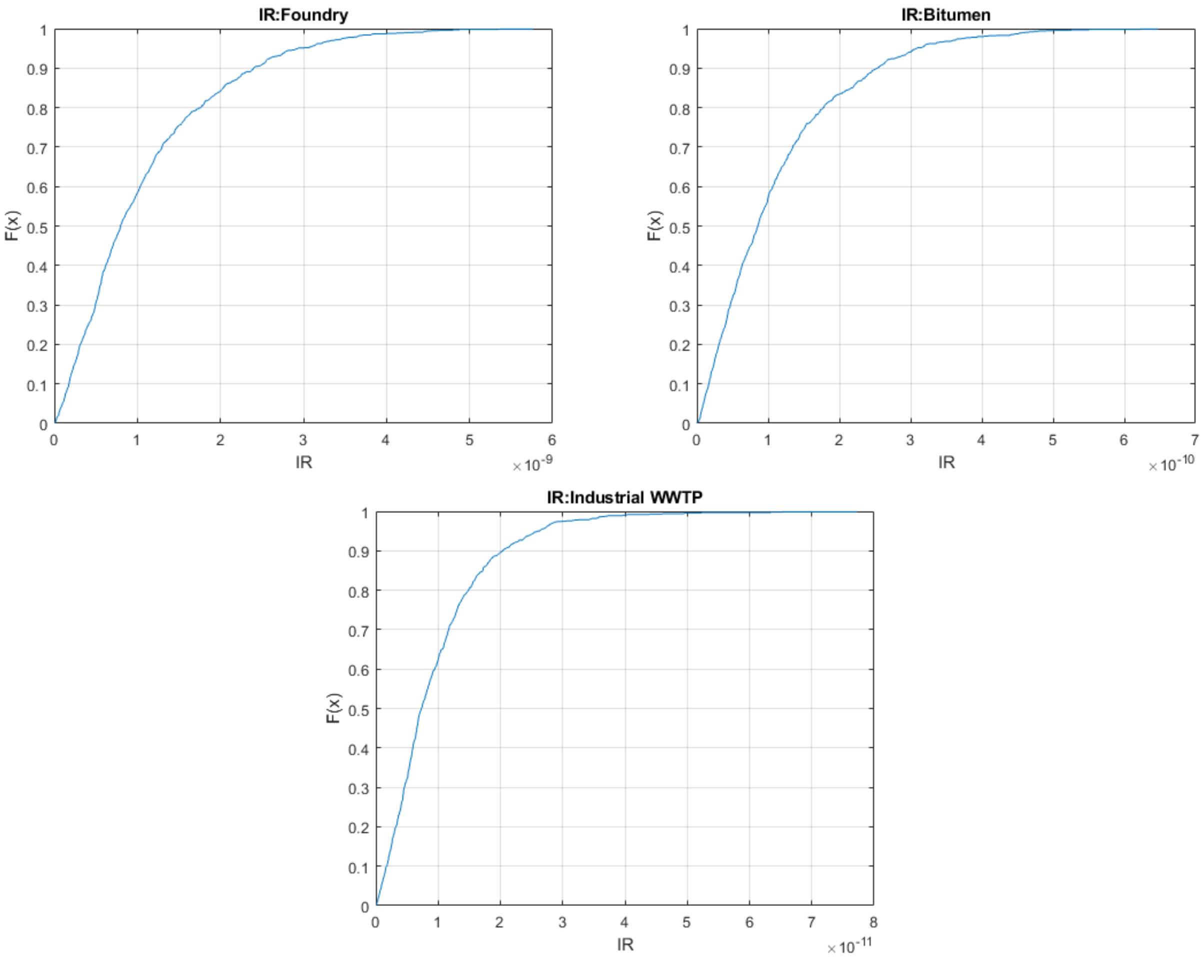

Figure S14. PDF of IR. Sample categories: *foundry, bitumen*, and *industrial WWTP*.

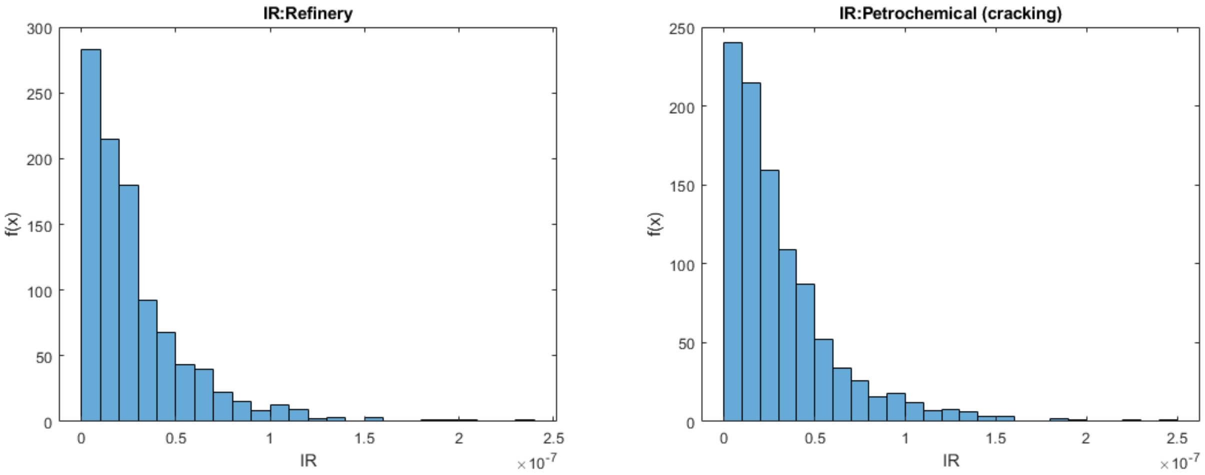

Figure S15. PDF of IR. Sample categories: *refinery* and *petrochemical (cracking)*.

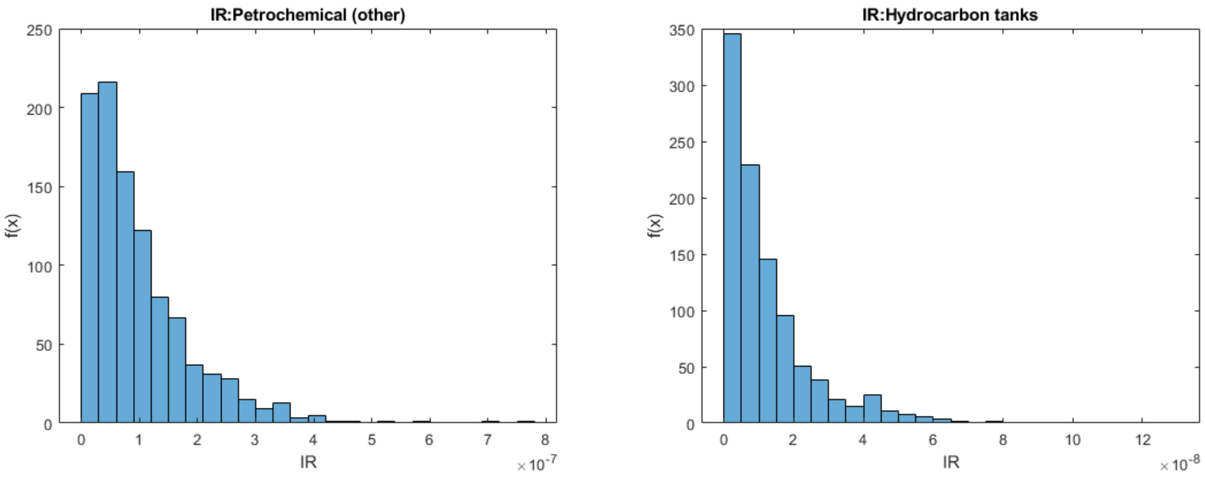

Figure S16. PDF of IR. Sample categories: *petrochemical (other)* and *hydrocarbon tanks.*

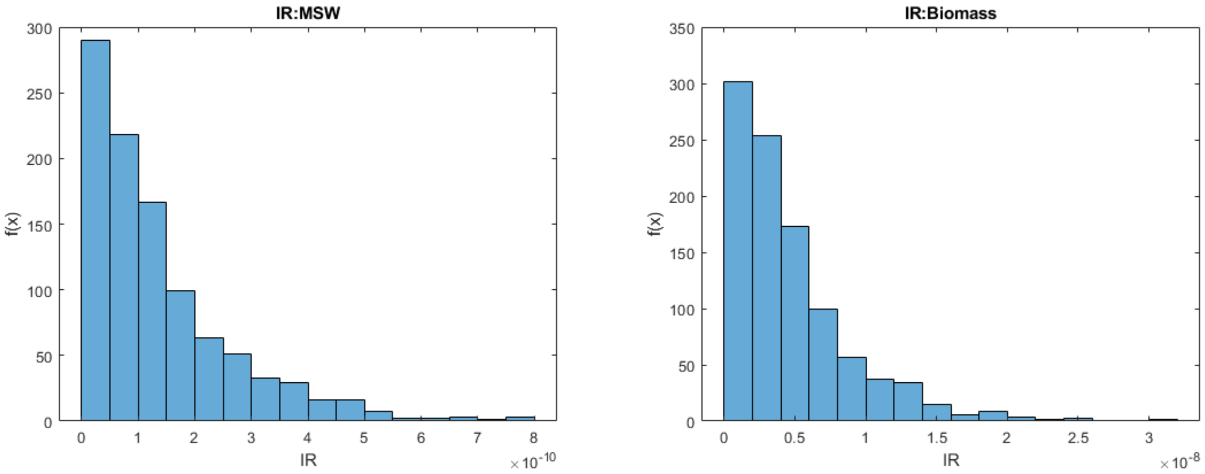

Figure S17. PDF of IR. Sample categories: *MSW* and *biomass.*

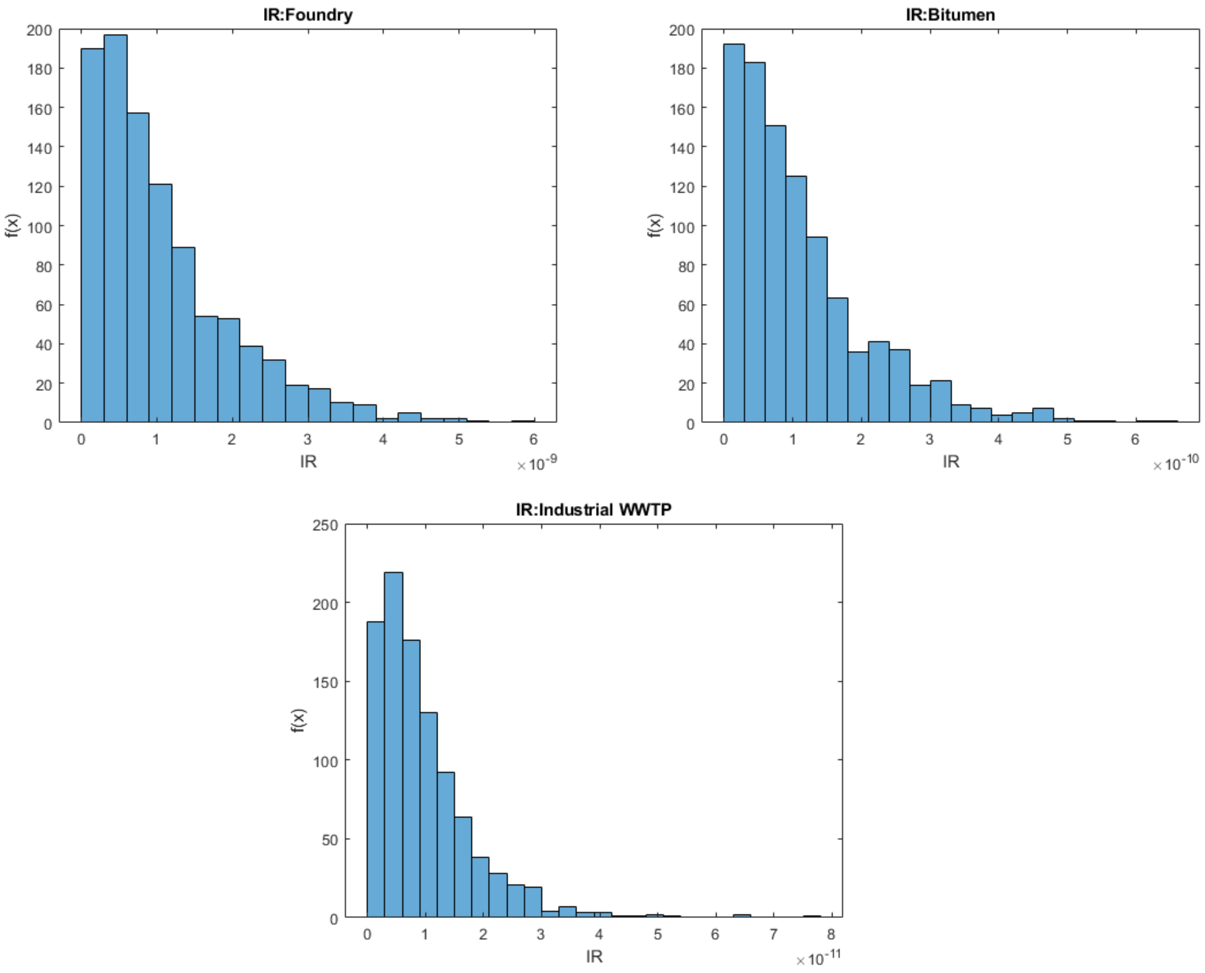

Figure S28. PDF of IR. Sample categories: *foundry, bitumen*, and *industrial WWTP*.
